# Novel Sterically Crowded and Conformationally Constrained α-Aminophosphonates with a Near-Neutral p*K_a_* as Highly Accurate ^31^P NMR pH Probes. Application to Subtle pH Gradients Determination in *Dictyostelium discoideum* Cells

**DOI:** 10.3390/molecules27144506

**Published:** 2022-07-14

**Authors:** Caroline Delehedde, Marcel Culcasi, Emilie Ricquebourg, Mathieu Cassien, Didier Siri, Bruno Blaive, Sylvia Pietri, Sophie Thétiot-Laurent

**Affiliations:** 1 Aix Marseille Univ, CNRS, ICR, UMR 7273, SMBSO, 13397 Marseille, France; caroline.delehedde@etu.univ-amu.fr (C.D.); marcel.culcasi@cnrs.fr (M.C.); emilie.ricquebourg@laposte.net (E.R.); bruno.blaive@univ-amu.fr (B.B.); sylvia.pietri@univ-amu.fr (S.P.); 2 Yelen Analytics, 10 Boulevard Tempête, 13820 Ensuès-la-Redonne, France; mathieu.cassien@yelen-analytics.com; 3 Aix Marseille Univ, CNRS, ICR, UMR 7273, CT, 13397 Marseille, France; didier.siri@univ-amu.fr

**Keywords:** α-aminophosphonates, pH probes, ^31^P NMR, phophorus pyramidalization angle, X-ray crystallography, trans-sarcolemmal pH gradients, *Dictyostelium discoideum* cells

## Abstract

In order to discover new ^31^P NMR markers for probing subtle pH changes (<0.2 pH unit) in biological environments, fifteen new conformationally constrained or sterically hindered α-aminophosphonates derived from diethyl(2-methylpyrrolidin-2-yl)phosphonate were synthesized and tested for their pH reporting and cytotoxic properties in vitro. All compounds showed near-neutral p*K*_a_s (ranging 6.28–6.97), chemical shifts not overlapping those of phosphorus metabolites, and spectroscopic sensitivities (i.e., chemical shifts variation Δ*δ*_ab_ between the acidic and basic forms) ranging from 9.2–10.7 ppm, being fourfold larger than conventional endogenous markers such as inorganic phosphate. X-ray crystallographic studies combined with predictive empirical relationships and ab initio calculations addressed the inductive and stereochemical effects of substituents linked to the protonated amine function. Satisfactory correlations were established between p*K*_a_s and both the 2D structure and pyramidalization at phosphorus, showing that steric crowding around the phosphorus is crucial for modulating Δ*δ*_ab_. Finally, the hit ^31^P NMR pH probe **1b** bearing an unsubstituted 1,3,2-dioxaphosphorinane ring, which is moderately lipophilic, nontoxic on A549 and NHLF cells, and showing p*K*_a_ = 6.45 with Δ*δ*_ab_ = 10.64 ppm, allowed the first clear-cut evidence of trans-sarcolemmal pH gradients in normoxic *Dictyostelium discoideum* cells with an accuracy of <0.05 pH units.

## 1. Introduction

Intracellular (cytosolic) pH (pH_i_) is a fundamental physiological parameter whose changes affect nearly all metabolic and growth/proliferation cellular processes, and can also occur in response to exogenous agents. Maintaining pH homeostasis in the cytosol, extracellular milieu (pH_e_) and subcellular compartments (such as lysosomes, mitochondria, and endosomal vesicles) is crucial for all living organisms. Among the mechanisms of pH_i_ regulation, the Na^+^/H^+^ antiporter represents a major pathway for the exit of protons from cells during acidification and the entry of Na^+^ from the extracellular medium to balance the net charge movement [1]. Indeed, pH_i_ and pH_e_ are in a dynamic steady state and, in normal mammalian cells, pH_i_ is close to neutrality (~7.2) while pH_e_ is slightly alkaline (ranging 7.3–7.5) [1]. Therefore, experimentally assessing pH regulation in pathological situations requires both pH_i_ and pH_e_ to be monitored simultaneously and accurately.

Since Moon and Richard’s first report [2], ^31^P nuclear magnetic resonance (NMR) has become a powerful and non-invasive technique for determining pH_i_ on the basis of the variation of pH, with the chemical shift of endogenous inorganic phosphate (PO_4_^3−^; P_i_) [3,4,5,6,7,8]. This technique has been applied to a variety of biological systems, e.g., for monitoring bioenergetics and/or the metabolism of various organs [9,10,11,12,13,14,15]. ^31^P NMR is also routinely used in the study of food chemistry, quality, and safety control [16,17] or for monitoring the production of everyday products [18], to name but a few. Despite P_i_ being ubiquitous in cells, its use as an ‘universal’ ^31^P NMR pH sensor in biological studies has, however, many drawbacks that may impair spectroscopic detection. First, given that the absolute value of the chemical shift difference between the protonated acidic (*δ*_a_) and unprotonated basic (*δ*_b_) forms of P_i_ (Δ*δ*_ab_ = |*δ*_a_ − *δ*_b_|) is only ~2.6 ppm, with a p*K*_a_ ~6.75 (for its second acidity [19]), it cannot sense pH_i_/pH_e_ differences of less than 0.3 pH units [2]. Moreover, the fluctuations of P_i_ concentration during metabolism and its low availability in the extracellular space and many organelles considerably hinder direct access to local pH measurements using this probe [20]. Ideally, the structural requirements for improved ^31^P NMR pH markers would both involve (i) increasing Δ*δ*_ab_ as to get pH titration curves with the steepest slope around the p*K*_a_ value and (ii) modulating the p*K*_a_ value(s) to fine tune extracellular/intracellular pH domains of interest.

**Figure 1:**
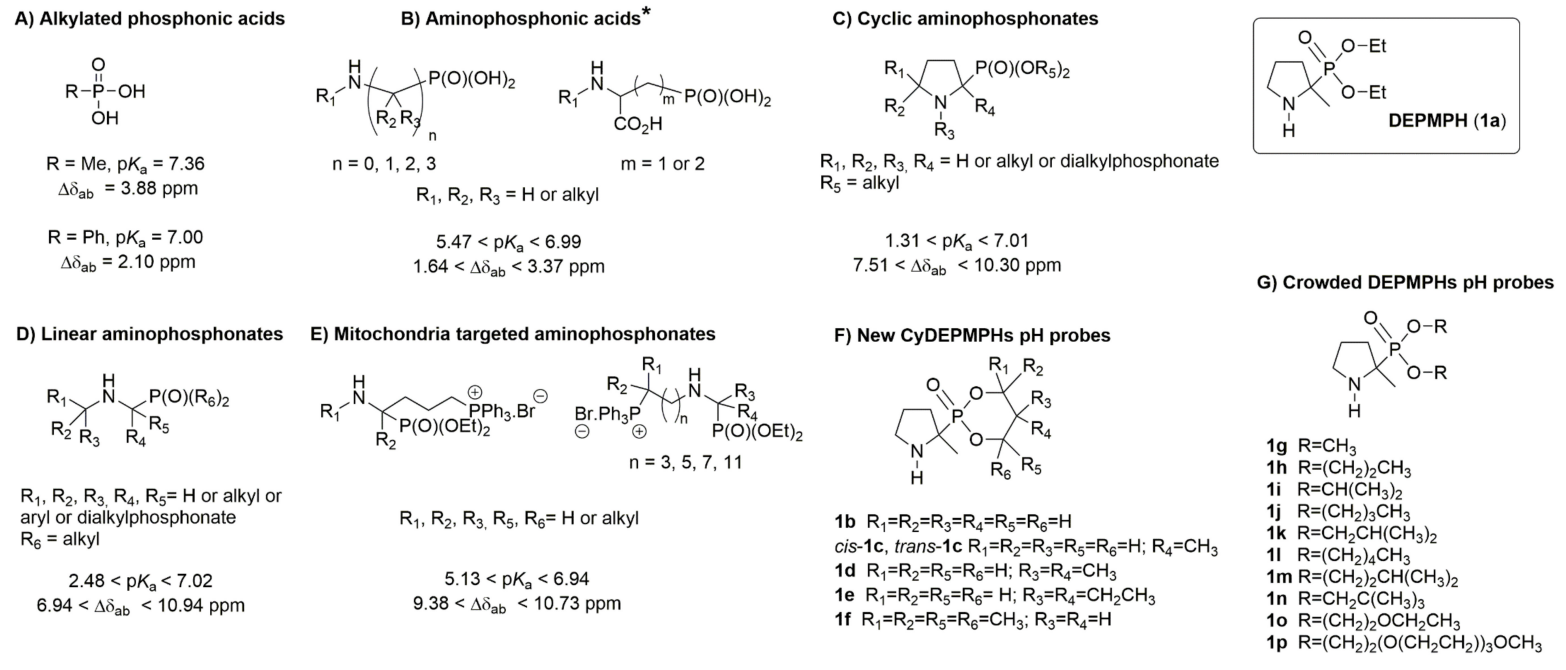
(**A**–**E**) Families of phosphorylated derivatives used as exogenous ^31^P NMR pH probes. (**F**) Conformationally constrained CyDEPMPHs pH probes (current study). (**G**) Sterically crowded DEPMPH-derived pH probes (current study). *Inset*: Structure of DEPMPH (**1a**). * p*K*_a_ and Δ*δ*_ab_ values of the second OH of phosphonic group.

In order to minimize the possible cytotoxic effects, the first synthetic attempts towards these above conditions focused on modifications around the P_i_ structure to get alkylated phosphonic acids RP (O)(OH)_2_ (Figure 1A), which were found to exhibit slightly increased Δ*δ*_ab_ values and p*K*_a_s of 7.0 ± 0.5 [21]. To illustrate, the methylphosphonate p*K*_a_ = 7.36, with Δ*δ*_ab_ = 3.88 ppm at 20 °C in a Krebs–Henseleit (KH) buffer is currently used in organ perfusion experiments [19]. Further targets were α-, β- and γ-substituted linear aminophosphonic acids (Figure 1B) whose Δ*δ*_ab_ values, however, did not exceed 3.4 ppm [22], but showed relatively weak toxicity and had lower p*K*_a_s suitable for probing the acidic domains in cells, tumors, and perfused organs [23,24,25,26]. Since in all these above compounds, protonation always occurs at the phosphorus first, it was then speculated that scaffolds bearing a non-ionizable *P*-atom (e.g., a phosphonate) and a protonable α-amino group (typically, a secondary amine) would offer considerable advantages in terms of Δ*δ*_ab_ and p*K*_a_ modulation due to different inductive and steric effects. This approach led to the development of two families of uncharged pH probes, including cyclic pyrrolidine-based α-aminophosphonates (Figure 1C) and linear polysubstituded α-aminophosphonates (Figure 1D) [19,27,28,29,30,31]. For all these compounds, it was found that protonation, which gives the ammonium salt as the acidic species, resulted in lower p*K*_a_s and gave Δ*δ*_ab_ values up to fourfold greater than that of P_i_ or phosphonic acids. Thus, the reported pH titration curves for the α-aminophosphonate having the structure shown in Figure 1D, with {R_1_ = R_2_ = R_3_ = Me; R_4_ = Et; R_5_ = Me; R_6_ = H} afforded Δ*δ*_ab_ = 10.3 ppm and p*K*_a_ = 7.01 in KH buffer [28]. Later, the successful mitochondrial targeting of selected compounds shown in Figure 1D was achieved by grafting a triphenylphosphonium cation (Figure 1E), and most of the resulting structures retained the good pH-probing performance of their parent compounds [32,33]. To illustrate, using a nontoxic micromolar concentration of the triphenylphosphonium bromide having the structure shown in Figure 1E (right part) with {R_1_ = R_2_ = H; R_3_ = R_4_ = Me; *n* = 7}, for which Δ*δ*_ab_ = 10.3 ppm and p*K*_a_ = 6.99, allowed the first ^31^P NMR assessment of acidic and cytosolic mitochondrial compartments of the green alga *Chlamydomonas reinhardtii* [33].

The knowledge of small trans-sarcolemmal pH gradients is one of the most important pieces of dynamic information to be obtained using very sensitive pH probes, especially when the external P_i_ peak is hardly or not detectable [34]. This was addressed in normoxic perfused rat hearts either by ^31^P NMR with the cell impermeable phenylphosphonic acid (Figure 1A; R = Ph) [34] or by ^19^F NMR using a fluorinated pyridine derivative [35], and in these early studies, reachable trans-sarcolemmal gradients were found to be 0.24 and 0.38 pH units, respectively. Based on their significantly larger Δ*δ*_ab_s when compared to, e.g., phenylphosphonic acid (having p*K*_a_ = 7.00 and Δ*δ*_ab_ = 2.10 ppm; Figure 1A [21]), many newer ^31^P NMR pH probes displayed in Figure 1C,D could be much better alternatives in probing more subtle pH changes (<0.2 pH units) occurring in acidotic cells. Actually, such an improvement was obtained using diethyl(2-methylpyrrolidin-2-yl)phosphonate **1a** (DEPMPH, having p*K*_a_ = 7.01 and Δ*δ*_ab_ = 9.77 ppm [19]; see Figure 1 inset) during ischemia of the rat isolated heart or liver [27]. However, despite the given advantage to probe the intra, extra and acidic regions simultaneously, **1a** was found to be less accurate than P_i_ for cytosolic pH determination [27]. The line width broadening at pH ≈ p*K*_a_ impairs any accurate pH determination [29], therefore excluding probes having a p*K*_a_ close to the pH region of interest. Moreover, the strictly neutral p*K*_a_ of **1a** was a drawback for assessing accurately the pH variations in acidic compartments [27]. It can therefore be inferred that, besides a Δ*δ*_ab_ value > 10 ppm, a better differentiation between the intracellular vs. extracellular NMR peaks would demand shifting of the p*K*_a_ of the probe downwards to, e.g., 6.2–6.8.

Previously a semi-empirical linear model was established for predicting the p*K*_a_ of **1a** and derivatives based on their 2D geometries and the additive inductive effect of substituents in β position of the nitrogen (e.g., R_1_–R_5_ and the phosphonyl group in Figure 1D) [19]. In the present study the DEPMPH pyrrolidine scaffold was used to elaborate novel derivatives in which the *P*-bound alkoxy groups were either constrained as a 2-oxo-1,3,2-dioxaphospharinane ring (CyDEPMPHs family **1b**–**f**; Figure 1F) or induced steric crowding around the *P*-atom (crowded family **1g**–**p**; Figure 1G). Compounds were investigated for their ^31^P NMR relaxation properties and pH reporting behavior in vitro. The collected experimental Δ*δ*_ab_ values and p*K*_a_s were found to be consistent inputs to consolidate the empirical relationships of [19]. Additional density functional theory (DFT) calculations were performed on the [C–P–O] pyramidalization angle of compounds **1a**–**p**, pooled with a large series of structurally related compounds. The results, together with structural complements using X-ray structures of those crystallized CyDEPMPHs, established a relationship between pyramidalization around P and Δ*δ*_ab_. From the above parameters, experimentally determined cytotoxicities, and calculated lipophilicities, the hit compound **1b** was selected and its ability to simultaneously probe the intra, extra and acidic regions was checked in normoxic *Dictyostelium discoideum* cells. For the first time, a trans-membrane pH gradient with an estimated precision of < 0.05 pH units was found in full accordance with the pH values given by the cytosolic and external P_i_ peaks, suggesting that **1b** could be a useful tool in a pathological situation where P_i_ peaks fall below the NMR detection limit.

## 2. Results and Discussion

### 2.1. Chemistry and X-ray Crystallography

**Scheme 1:**
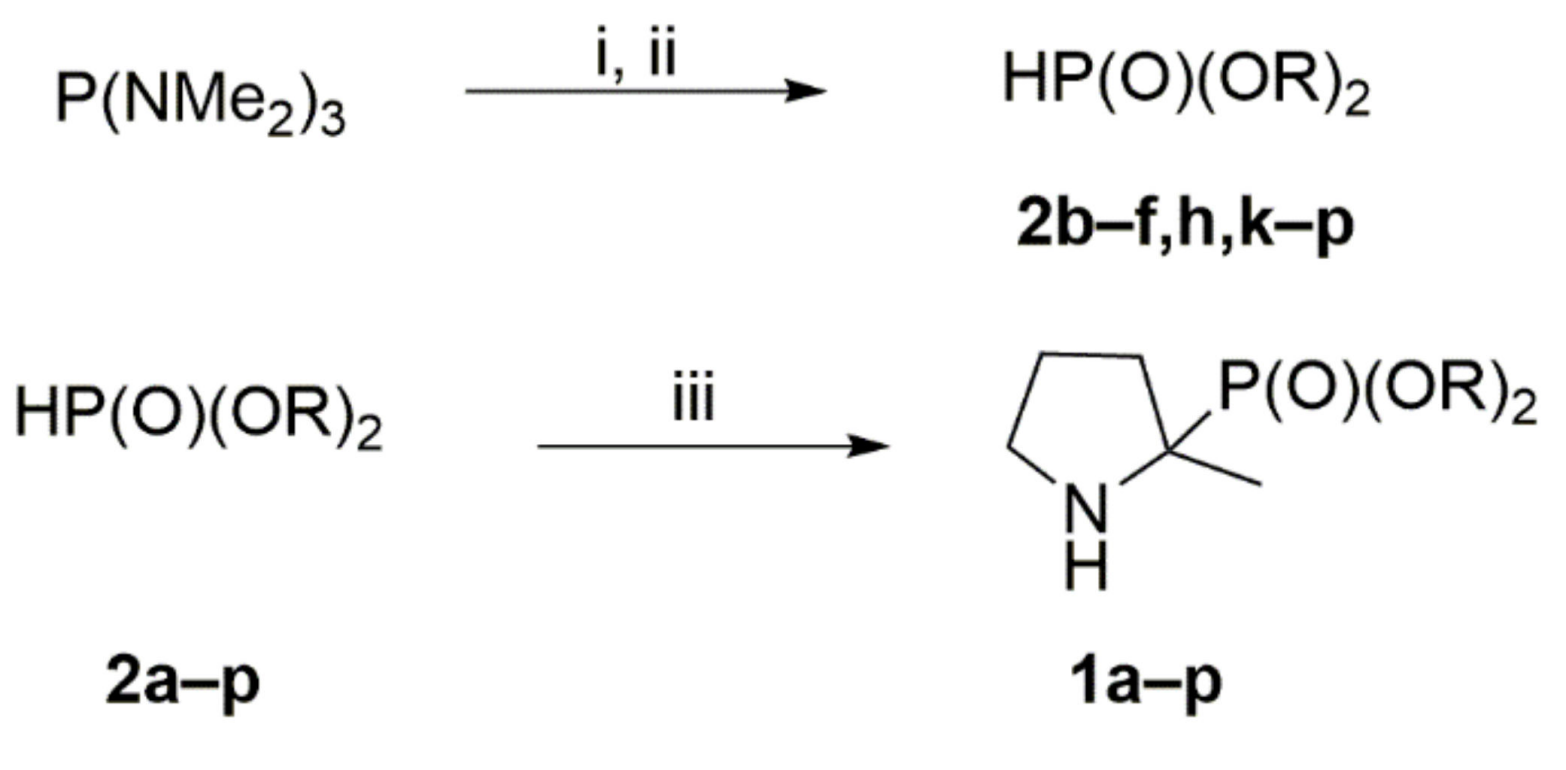
General synthesis of aminophosphonates **1a**–**p**. Reagents and conditions: (i) 1 equiv H_2_O, 3 h, 70 °C, THF; (ii) 1 equiv diol (series **b**–**f**), 6 h, 70 °C, THF or 2 equiv alcohol (series **h**,**k**–**p**), 3 h, 70 °C, THF; (iii) 1.1 equiv 2-methyl-1-pyrroline, 2 h–10 d, rt, toluene.

Aminophosphonates **1a**–**p** were synthesized according to the general sequence outlined in Scheme 1, involving the reaction of the corresponding dialkyl H-phosphonate **2a**–**p** with 2-methyl-1-pyrroline. Starting compounds dialkyl H-phosphonates **2a**, **2g**, **2i**, and **2j** are commercially available, while the other precursors **2b**–**f**, **h**, **k**–**p** were synthesized according to [36] with slight modifications. Briefly, H-phosphonic bis(dimethylamide) was prepared in situ by the addition of one equivalent of water to hexamethylphosphorous triamide (HMPT) in tetrahydrofuran (THF), and then alcohol was added to give the corresponding compounds **2b**–**f**, **h**, **k**–**p**. Reactions were monitored directly by ^31^P NMR by following the disappearance of HMPT peak (*δ* = 122.24 ppm) followed by the formation of bis(dimethylamino)phosphoric acid peak (*δ* = 24.0 ppm), then by the disappearance of this latter and the formation of the corresponding dialkyl H-phosphonate peak. Dialkyl (2-methylpyrrolidin-2-yl) phosphonates **1a**–**p** were obtained by the nucleophilic addition of dialkyl H-phosphonates **2a**–**p** onto 2-methyl-1-pyrroline in toluene [37] in good yields (49–73%).

**Figure 2:**
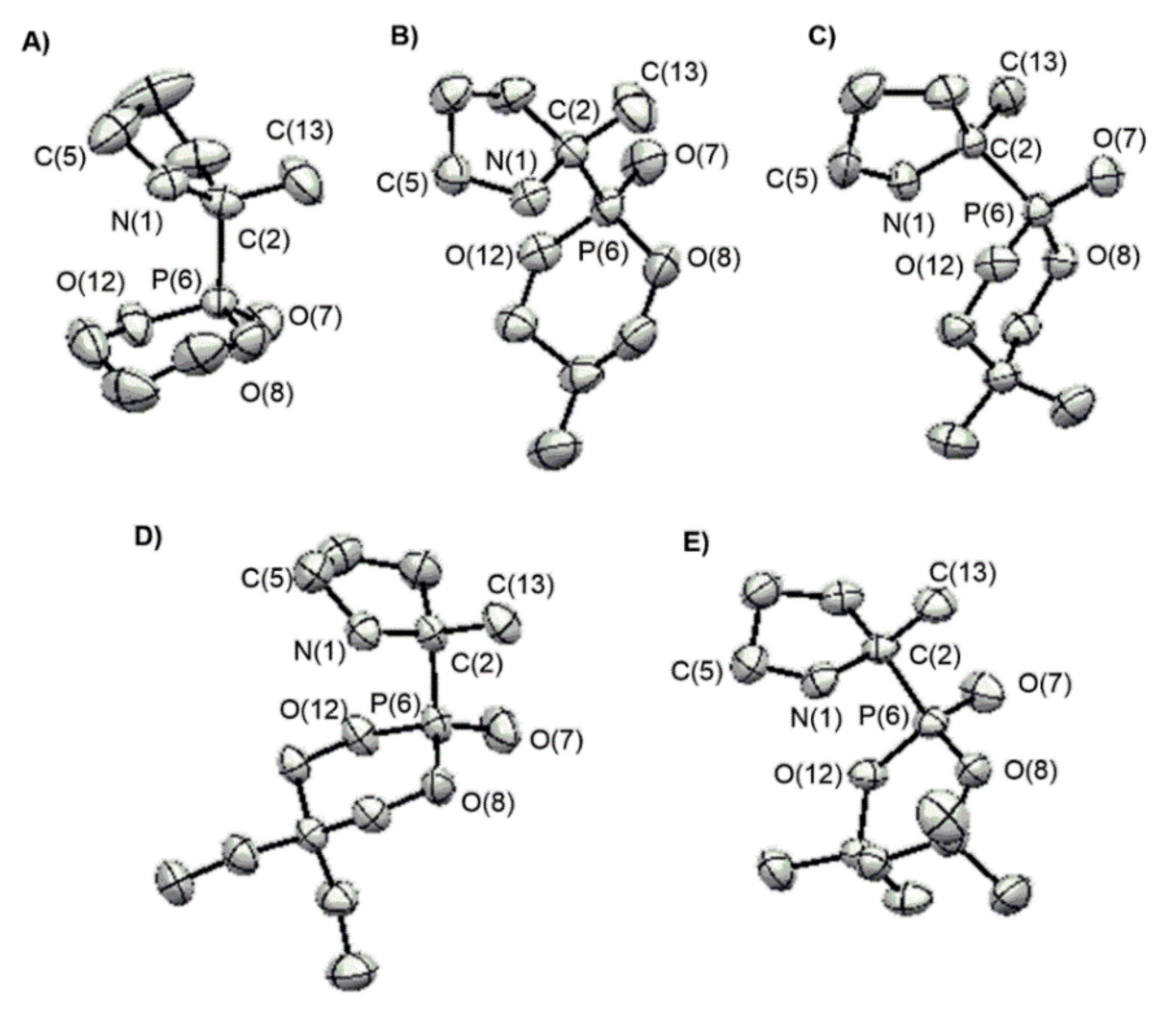
ORTEP drawings of CyDEPMPHs (**A**) **1b**; (**B**) *trans*-**1c**; (**C**) **1d**; (**D**) **1e**; (**E**) **1f** showing the labeling of atoms (see also Table 1 heading) and their displacement ellipsoids at the 40% probability level. For clarity, H-atoms have been omitted.

Single crystals suitable for X-ray diffraction analysis were obtained for the five compounds **1b**, *trans*-**1c**, and **1d**–**f** by recrystallization from a dichloromethane:*tert*-butylmethylether (TBME) mixture (Figure 2). The pyrroline rings were found to adopt puckered conformations which can be either the E + 4 (ϕ = 72°) envelope conformation (**1b**), the T1 (ϕ = 18°) twist conformation (*trans*-**1c**) or an intermediate situation between the T1 and E + 4 conformations (**1d**–**f**) according to the classification of [38]. The C–P bond is pseudo-axial in compounds **1e** and **1f** and pseudo-equatorial in compounds **1b**, *trans*-**1c**, and **1d** (Table 1), as referred to by the sign of the C(5)–N(1)–C(2)–C(3) dihedral angle in nitrones analogues of CyDEPMPHs [39].

### 2.2. pH Dependent ^31^P NMR Properties of New α-Aminophosphonates

**Figure 3:**
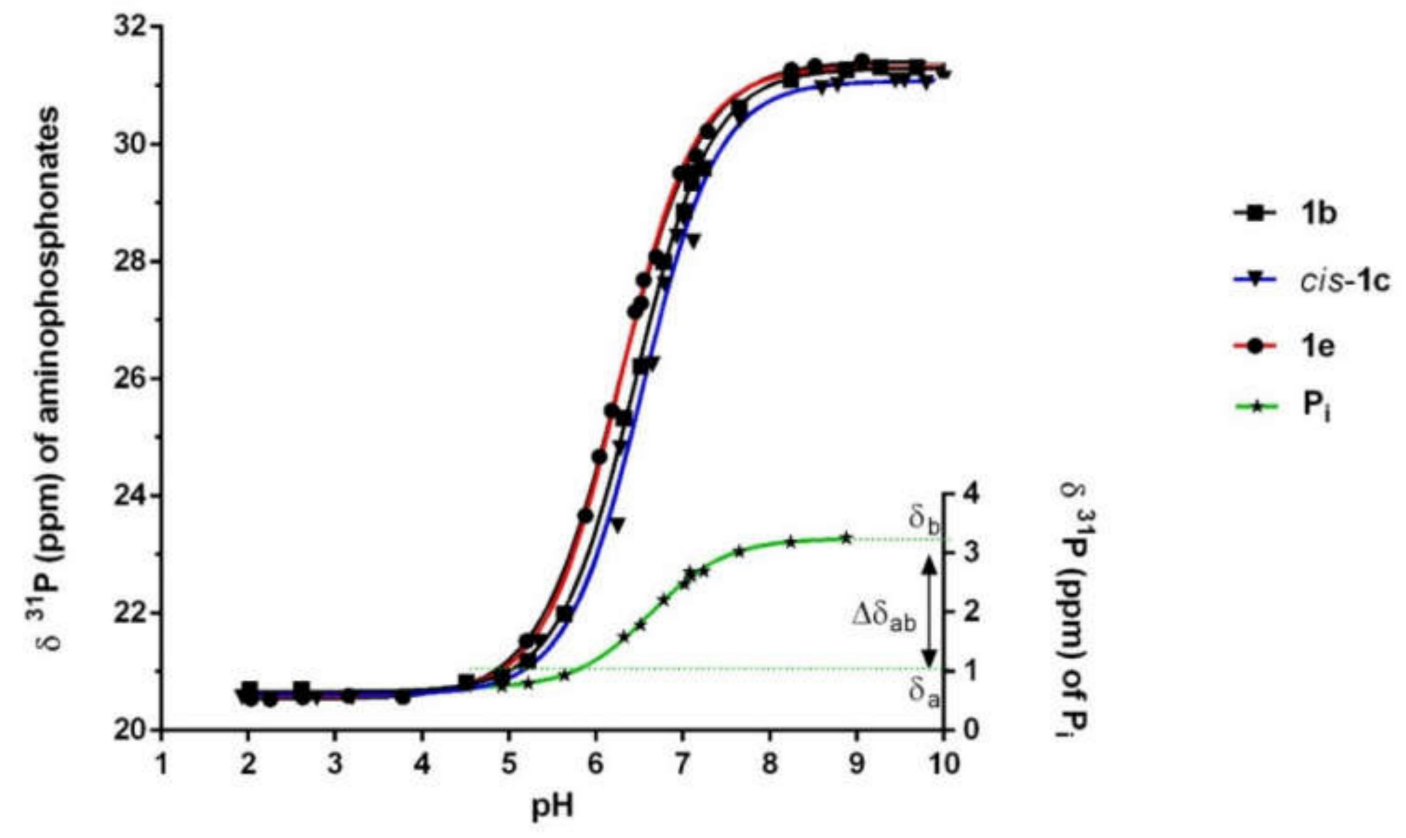
pH Dependence of the ^31^P chemical shift of CyDEPMPHs **1b**, *cis*-**1c**, and **1e** compared to P_i_. Titrations were performed at 22 °C in a modified KH buffer (pH 7.35) and each curve was obtained by the nonlinear fitting of 3–10 independent experiments. Outlined: limiting acidic (*δ*_a_) and basic (*δ*_b_) chemical shifts, and their difference (Δ*δ*_ab_) for P_i_.

The pH dependence of the ^31^P NMR chemical shift of **1a**–**p** was investigated at 22 °C in KH buffer (pH 7.35). Monophasic acid–base titration curves reflecting protonation on nitrogen were obtained for all the derivatives (see examples in Figure 3) and their fitting to the Henderson–Hasselbalch equation (see Equation (2) in Section 3) allowed the calculation of p*K*_a_s, the ^31^P NMR parameters (*δ*_a_, *δ*_b_, and Δ*δ*_ab_), and the spin lattice relaxation time (*T*_1_) (Table 2).

All the new derivatives fulfilled both the desired conditions for more accurate pH probing (see Introduction), exhibiting near-neutral p*K*_a_s (ranging 6.28–6.97) and very high Δ*δ*_ab_ values. Indeed, the average ^31^P NMR sensitivities obtained here could compete with those of the best linear aminophosphonates available so far (Table S1). Interestingly, the compounds having no constrained phosphorus bound alkoxy groups such as **1a** and almost all elements from the crowded family showed slightly more basic p*K*_a_s and lower ^31^P sensitivities when compared to compounds from the CyDEPMPHs family, for which Δ*δ*_ab_ > 10 ppm. The only exception was compound **1f,** which retained a high sensitivity despite Δ*δ*_ab_ = 9.35 ppm and a p*K*_a_ value in the range of that of the crowded DEPMPHs (Table 2).

In a biological milieu where NMR peaks are usually broad, any added pH probe should exhibit protonated and non-protonated forms in fast exchange on the NMR timescale to provide a single sharp peak for each cytosolic/intracellular compartment. This implies that the *T*_1_ time of the probe should be as short as possible in order for the best resolved signals to be recorded within the timescale of expected variations of biological parameters, such as pH [28]. All new pH probes demonstrated *T*_1_ values similar to those found in other related aminophosphonates [28,30]. Expectedly, molecular motion restriction in the conformationally constrained CyDEPMPHs **1b**–**f** versus the long-chain linear phosphonates **1l**–**p** from the crowded series generally resulted in slightly increased *T*_1_ values (Table 2). It is worth noting that probe exchanges between cell compartments are slow on *T*_1_ and chemical shift NMR timescales, while diffusion kinetics may exhibit strong variations for linear versus cyclic aminophosphonates (e.g., being 10 min for **1a** and 60 min for 2-aminoethylphosphonate (Figure 1B with R_1_–R_3_ = H and *n* = 2) [29]). Given that pH_i_ variations in vivo may occur in minutes (reported value were of ~10 min following acidosis [40] and 15–20 min following alkalinization [41]), *T*_1_ does not therefore appear to be a decisive parameter to discriminate between probes having similar chemical structures.

### 2.3. pKa Modeling as a Function of Substituents

Predicting p*K*_a_ values by molecular modeling is of paramount importance in the design of improved ^31^P NMR pH markers. In this regard, a pertinent approach is to consider the pH-dependent variations of electron density around the protonation site; that is, p*K*_a_ should depend linearly on the sum of individual inductive and stereochemical contributions of substituents. This has been applied successfully, first to a series of alkylphosphonic acids as depicted in Figure 1A [42], second to the cyclic and linear α-aminophosphonates developed in the early 2000s (see Figure 1C,D). In these studies [19,28], p*K*_a_s were computed using the semi-empirical linear model of Equation (1):

p*K*_a_ = *a*_0_ + ∑*a*_i_ × *n*_i_ (1)

where, for a given structurally related family of compounds, *a*_0_ is the p*K*_a_ of the common parent chemical structure and parameters *n*_i_ (with maximum Σ*n*_i_ = 6) and *a*_i_ (in pH units, with 1 < *a*_i_ < 10) represent the number of each different kind of substituents of type *i* having the same structure and/or position relative to the nitrogen protonation site and the weight of its electronic effect relative to the p*K*_a_ value *a*_0_, respectively.

**Figure 4:**
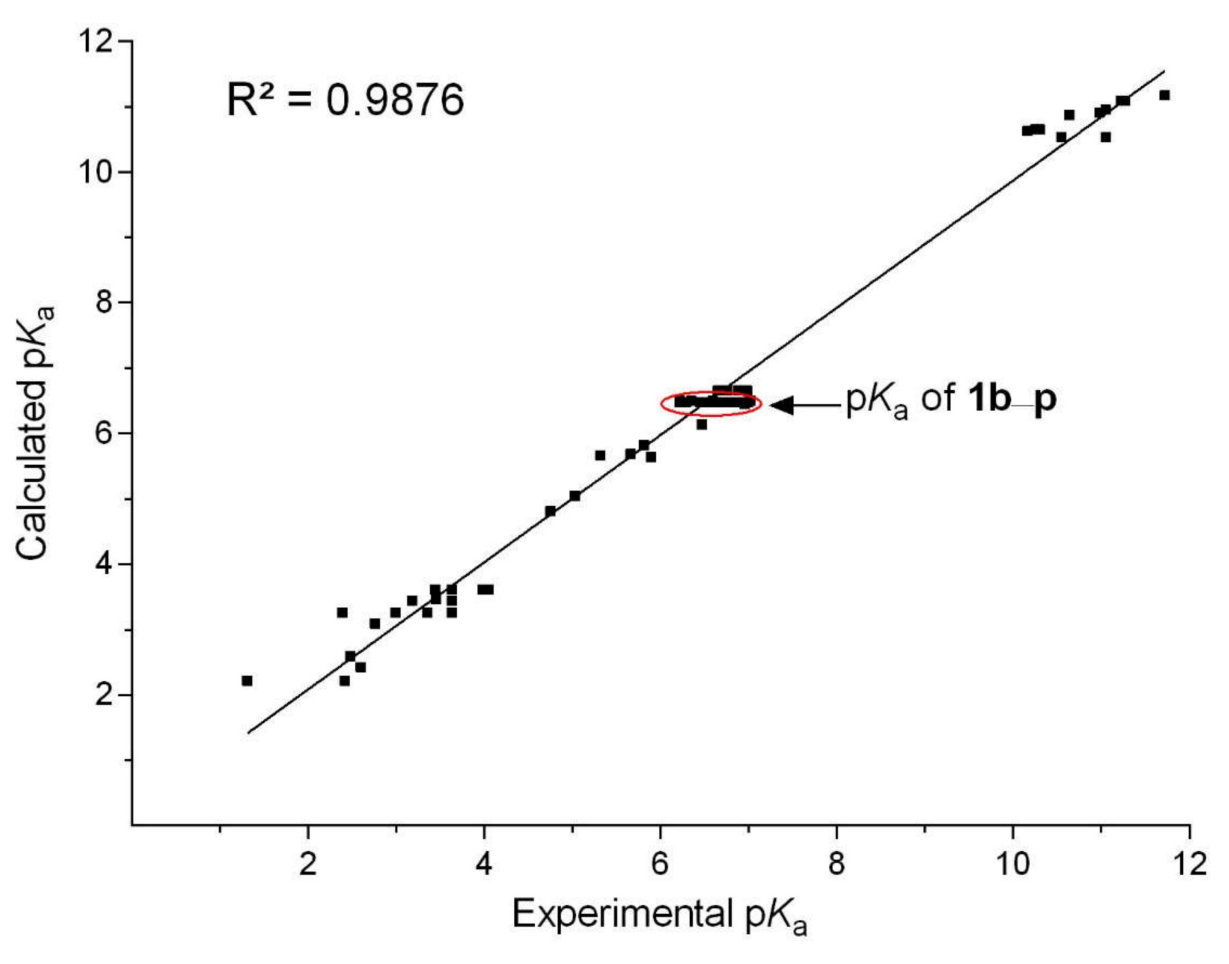
Correlation between experimental and calculated p*K*_a_ values for a pooled database of 59 α-aminophosphonates using the updated linear predictive model of Equation (1) (see also Table S2). The red circle indicates the p*K*_a_ domain of new compounds **1b**–**p**.

Starting from the general dialkylamino structure with up to seven surrounding substituents, a new refinement, including compounds **1a**–**p**, of the previous *a*_i_ database collected from earlier studies afforded the updated Table S2 (see general structure in heading), where the selected reference structure bears a cycloalkyl substituent. The extended *a*_i_ database encompassed a total of 59 aminophosphonates, comprising **1a** and the new analogues **1b**–**p**, twenty-two linear (LAP-**1**–**22**) and ten cyclic (CAP-**1**–**10**) aminophosphonates, five aminophosphophonic acid derivatives (APA-**1**–**5**), and six alkylamines (Tables S1 and S2) studied previously [19,22,28,29,30,31,39,43,44]. As shown in Figure 4, updating the *a*_i_ values still resulted in a good fit (R^2^ = 0.9876) between experimental and calculated p*K*_a_s.

In the computational p*K*_a_s model of Equation (1), all compounds **1a**–**p** share the same substitution pattern, i.e., one H atom on the nitrogen, two C(5)–H, three alkyls on C(1) and C(5), and one C(1)–Phosphonate. Having the same {*n*_1_–*n*_10_} set of {1, 0, 2, 3, 0, 0, 0, 0, 1, 0}, compounds **1a**–**p** obviously yielded the same calculated p*K*_a_ = 6.475, while an experimental range of Δp*K*_a_ = 0.75 was found (Table 2). When compared to this common p*K*_a_ value, the experimental p*K*_a_s of the more substituted compounds **1f** and **1i** were found to be strongly underestimated by 0.315 and 0.495 units, respectively (Table 2), suggesting that steric factors should be accounted for in the p*K*_a_s predictive model.

### 2.4. [C–P–O] Pyramidalization Angle Calculations

Previously, electrostatic interactions such as internal ^+^NH_2_····(O)P hydrogen bonding have been proposed to explain the shielding of the NMR peaks occurring upon protonation of α-aminophosphonates (i.e., *δ*_b_ > *δ*_a_), an effect that would decrease the pyramidalization of the angle [C–P–O] of the tetrahedral phosphorylated moiety [19]. In view of the lack of accuracy of the predictive model of Equation (1) for the most sterically hindered new pH probes, it was investigated whether additional conformationally derived effects may be implicated.

**Figure 5:**
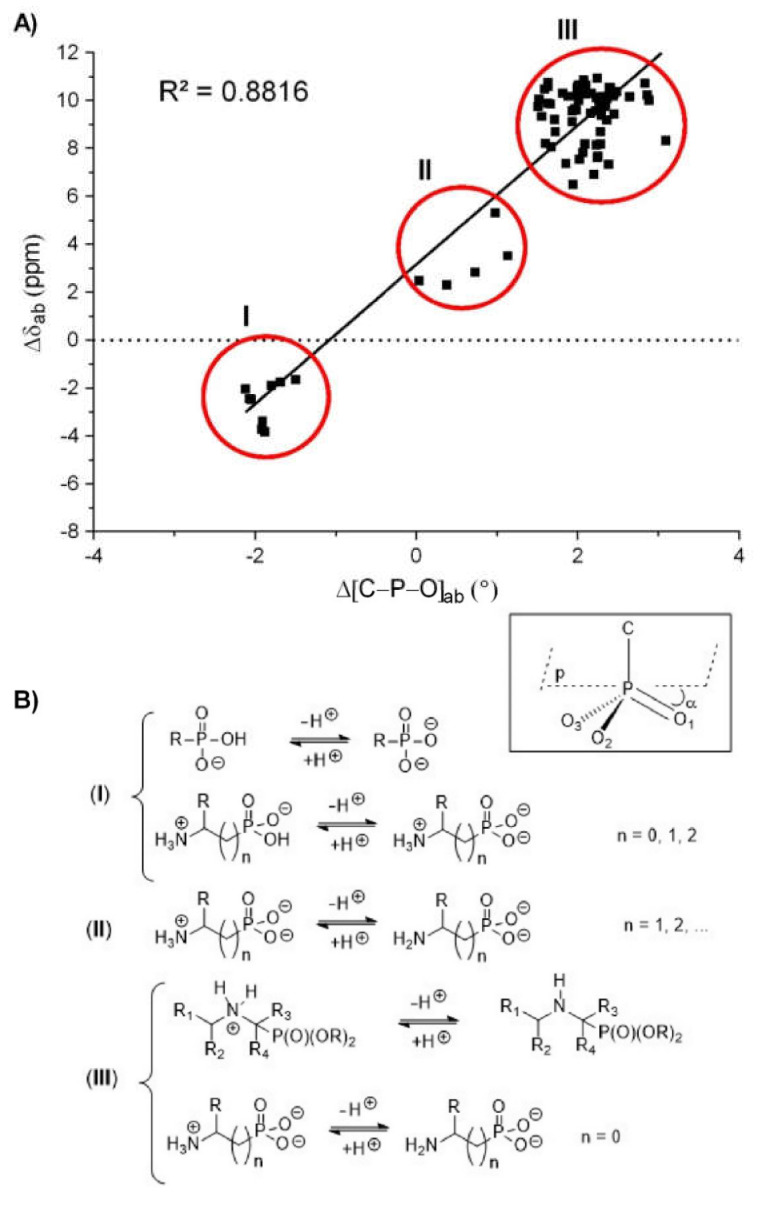
(**A**) Plot of ^31^P NMR sensitivity against ab initio calculated pyramidalization angles at phosphorus between the acidic and basic forms of a series of phosphorylated pH probes. Circled regions I to III visualize increasing pH probing properties. (**B**) Proton exchange reactions on (I) the O atom of phosphonic or aminophosphonic acids, (II) the N atom of aminophosphonic compounds with at least two carbons between the N and P atoms and (III) the N atom of aminophosphonates or aminophosphonic acids with only one carbon between the N and P atoms. *Inset*: Pyramidalization angle *α*.

DFT-B3LYP calculations using the Gaussian16 package were carried out for optimizing [C–P–O] angle variations between acidic and basic forms (Δ[C–P–O]_ab_) for **1a**–**p**, pooled with the previously studied compounds LAP-**1**–**34**, CAP-**1**–**10**, and APA-**1**–**7** (Table S1) [19,22,28,29,30,31,32,39,43,45]. For this large set of compounds (corresponding to 78 pairs of acidic and basic chemical shifts) a moderately good linear correlation (*y* = 2.907 *x* + 3.150; R^2^ = 0.8816) was obtained between Δ[C–P–O]_ab_ and Δ*δ*_ab_ (Figure 5A), confirming that pyramidalization at phosphorus should have a notable impact on the ^31^P NMR sensitivity of aminophosphonates. For simplification, the [C–P–O] pyramidalization angle α here was defined as the average of the three angles between P–O_1–3_ bonds and the *p*-plane orthogonal to the P–C bond (Figure 5B, inset); that is, α decreases as the pyramid is more flattened. Negative Δ[C–P–O]_ab_ variations associated to Δ*δ*_ab_ < 0 (i.e., *δ*_a_ > *δ*_b_) were found for APA-**1**–**6** aminophosphonic derivatives (Table S1) in the p*K*_a2_ region of the second acidity (Figure 5A, region **I**, and Figure 5B, reactions I). A second group of molecules (Figure 5A, region **II**, and Figure 5B, reactions II) showed small changes in the pyramidalization angle (0 < Δ[C–P–O]_ab_ < 1.13°), associated with low ^31^P NMR sensitivities (2.3 < Δ*δ*_ab_ < 5.3 ppm). These latter structures bear at least two carbons between the N and P atoms of the phosphonic derivatives (LAP-**23**, APA-**2**,**5**–**7**; Table S1).

As anticipated behind the design of compounds **1b**–**p**, Figure 5A (region **III**) and Figure 5B (reactions III) show that those molecules having the largest Δ[C–P–O]_ab_ (ranging 1.51–3.60°) also exhibited the greater Δ*δ*_ab_s (6.48 < Δ*δ*_ab_ < 10.94 ppm). Together with **1a**–**p**, region **III** compounds in Figure 5A also included the derivatives APA-**1**, LAP-**1**–**22**,**24**–**34**, and CAP-**1**–**10** (Table S1); that is, in all these molecules, the environment around P is moderately to strongly sterically crowded. Of interest, among the CyDEPMPHs, the apparently less constrained **1b** (Figure 2A), which bears a less bulky unsubstituted 1,3,2-dioxaphosphorinane ring, demonstrated the highest protonation-induced Δ[C–P–O]_ab_ value of 2.10°.

### 2.5. In Vitro Cytotoxicity Studies

The cytotoxic activities of compounds **1a**–**p** in A549 human lung carcinoma cells and in normal human lung fibroblasts (NHLF) were evaluated in 0.2% DMSO-supplemented Dulbecco’s modified Eagle’s medium (DMEM) and fibroblast basal medium (FBM), respectively. The methods used included the tetrazolium dye MTT reduction, the fluorometric microculture cytotoxicity (FMCA), and the intracellular ATP assays. The MTT and FMCA assays are standard colorimetric determinations of cell viability during in vitro drug treatment while the ATP assay is an index of metabolic activity.

The IC_50_ values following 48 h incubation at 37 °C of the cells with varying concentrations of test compounds are reported in Table 3, together with the predicted lipophilicities based on AlogP calculations. When the three above-cited endpoints were found to be affected by treatment with a test compound, the release of cytosolic lactate dehydrogenase (LDH) was assessed to check for cell necrosis.

In the supernatant of untreated A549 and NHLF cells, the baseline LDH value was found to be 10.2 ± 1.5 and 11.3 ± 1.9 UI/mg protein, respectively. When compared to the total LDH activity measured after 100% lysis of the cells, which was 690 ± 47 and 701 ± 34 UI/mg protein for A549 and NHLF, respectively, the baseline released LDH vs. total cellular LDH in untreated cells was found to be as low as 1–2%.

After 48 h incubation of the cells, no significant dispersion among assays was found with compounds **1b**–**e** of the CyDEPMPHs family (*p* > 0.05 by one-way ANOVA), suggesting those aminophosphonates induced cell death by altering metabolic activity, enzymatic and mitochondrial functions rather than by causing membrane damage and/or cell necrosis. The more lipophilic **1d** and **1e** were found to be slightly more toxic (Table 3).

Regarding the three viability assays, the more lipophilic compound of the CyDEPMPHs family, **1f**, was associated with the strongest global decrease of IC_50_ values, which was of 42–50% (*p* < 0.05) as compared to **1b**, and reached 50–60% (*p* < 0.05) as compared to **1a**. In parallel, the application of **1f** resulted in a significant LDH leakage (>45% of total baseline LDH content; *p* < 0.01 vs. untreated cells and **1a, 1b**) showing it additionally caused cell necrosis.

In the crowded family of pH probes, the decrease in viability globally paralleled the increase of lipophilicity, except for **1o** and **1p**. Hence, IC_50_ values for **1j**–**p** decreased by 60–80% (*p* < 0.05) as compared to the hydrophilic **1g** in the MTT and ATP assays, while an even greater impact was found for these lipophilic compounds in the FMCA assay (i.e., a 80–90% decrease; *p* < 0.05 vs. **1g**), suggesting they induced membrane damage at concentrations > 10 mM (Table 3). Consistently, application of **1j**–**p** to both types of cells resulted in a strong LDH leakage at IC_50_ values (60–80% of total baseline intracellular content).

Altogether, the cytotoxicity data of Table 3 demonstrated that most of the new pH probes, i.e., **1b**–**e** and **1g**–**i**, could be added safely in cell medium at a concentration window (up to 1–20 mM) compatible with NMR applications. Due to its innocuity and optimal ^31^P NMR titration parameters (p*K*_a_ = 6.45; Δ*δ*_ab_ = 10.64 ppm), compound **1b** was considered ideal for probing small pH gradients, since it can be used at 4 mM, a concentration at which >98% cell viability was preserved.

### 2.6. Application of the ^31^P NMR pH Probes ***1b*** vs. ***1a*** in Dictyostelium discoideum Amoebae

*Dictyostelium discoideum*, a widely used cell model for investigating acidic organelles, has been found to be particularly suitable to monitor by ^31^P NMR the kinetics of anoxia- and reoxygenation-induced pH variations in various endosomal acidic vesicles [23]. In the search for improved resonance tools for assessing trans-sarcolemmal pH gradients, i.e., pH_i_/pH_e_ differences, *D. discoideum* was also found to be a reliable model, since in this case endogenous and external ^31^P NMR P_i_ peaks are easily distinguishable [21] and may allow a direct comparison with the expected resonances arising from the probe, provided it could significantly accumulate within the cytosolic and external compartments. Such properties were not reported in other models, including the rat heart [46] and liver [47] due, among other reasons, to the low and/or varying concentrations of P_i_, the large line widths of cellular ^31^P NMR peaks, and the low pH_i_/pH_e_ difference < 0.5 pH units.

**Figure 6:**
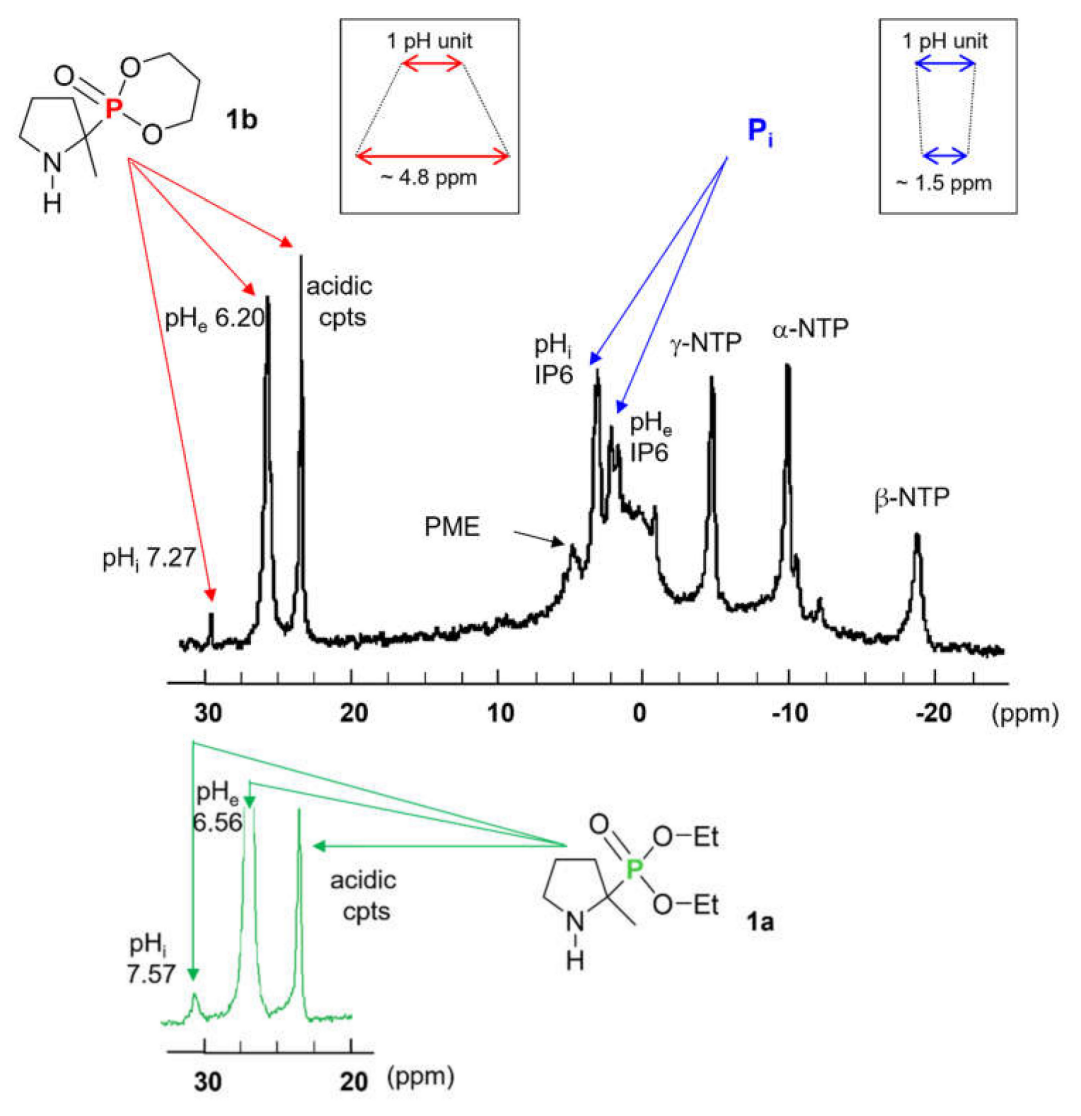
^31^P NMR spectra from *D. discoideum* cells (3 × 10^8^ cells/mL) aerobically incubated at 20 °C in the presence of 4 mM of **1b** (upper trace) or **1a** (green trace, only deshielded region shown). The signals correspond to a 15-min accumulation (900 scans) starting 15 min after addition of the probe. Using the corresponding titration curves allowed for the simultaneous calculation of all pH values, i.e., intracellular (pH_i_), extracellular (pH_e_), and that of acidic compartments (cpts). Cytosolic and extracellular inorganic phosphate (P_i_) pH values (pH_i_ ~7.24 and pH_e_ ~6.32) were drawn from the **1b** titration curve (Figure 3). Shielded resonances in the upper trace are from phosphomonoesters (PME); inositol hexaphosphate (IP6); γ-, α-, and β-nucleoside triphosphates (NTPs) [21,29]. Chemical shifts are given in ppm with respect to 85% external H_3_PO_4_ at 0 ppm and to 50 mM methylene diphosphonate (pH 8.9) placed in a capillary and used as an additional standard at 16.4 ppm. *Insets*: a schematic representation for comparison between **1b** and P_i_ pH probing accuracies around their p*K*_a_ values.

Figure 6 shows representative ^31^P NMR spectra obtained during incubation at 20 °C of normoxic amoebae in the presence of 4 mM of **1a** (lower trace, deshielded region) or **1b** (upper trace). In the more deshielded spectral region (20–30 ppm) of the experiment with **1b**, prominent resonances were apparent, likely arising from the probe internalized in cells and located in three distinct intracellular compartments. From the calibration curve of **1b** (Figure 3), the corresponding pH values were calculated as ~5.6 (for the more acidic compartment at 22.6 ppm) and 6.20 and 7.27 for the lowfield peaks at 25.4 and 29.9 ppm, respectively. These two latter compartments probed by **1b** likely correspond to the extracellular and cytosolic environments, in full accordance with the pH values determined by the two P_i_ peaks, pH_i_ ~7.24 (cytosol) and pH_e_ ~6.32 (extracellular milieu).

When **1a** was used instead of **1b,** acidic compartments were similarly probed at 23.6 ppm (pH~5.7) while the cytosolic and extracellular regions exhibited broader lines and higher apparent pH values of pH_i_ 7.57 (resonance at 30.5 ppm) and pH_e_ ~6.56 (broad resonance at ~26.5 ppm), respectively. This showed that **1a** accumulated mainly in the external space vs. the cytosol and confirmed the previous observations [29] of (i) a line broadening at the resonance corresponding to the cytosolic pH, and (ii) a slight alkalinization of the cytosolic and extracellular compartments because of the higher p*K*_a_ of the probe, which behaved as a weak base.

The innocuity of **1b** (4 mM) in the system was confirmed by its lack of effect on the endogenous intracellular phosphorylated compounds including nucleotides, inositol hexakisphosphate and phosphomonoesters, as compared to control spectra (data not shown). As already reported with other series of cyclic and linear α-aminophosphonates [29], **1b** was internalized in amoebae in less than 5 min (data not shown) both in the cytosol, extracellular region and acidic vesicles. To explain the rapid rate of diffusion and intracellular distribution observed for **1b** and other aminophosphonates [27,29], it was supposed that these compounds are membrane permeable under their unprotonated amine form, and can accumulate by passive diffusion [29]. This general property confers a great advantage to α-aminophosphonates as compared to the commonly used alkylated phosphonic acids derivatives, which were shown to internalize slowly, at least during 90 min, within the cells, involving energy requiring processes such as continuous H^+^ pumping by vacuolar H^+^–ATPase, leading to NTP depletion [48].

To stress on the specific ^31^P NMR pH reporting properties, the biocompatible **1b** showed a ~3.2 better potential accuracy than P_i_ (Figure 6). This may allow us to reliably address pH differences as low as 0.05 pH units, corresponding to narrow peaks separated by ~0.25 ppm. As anticipated in the design of the novel pH probes, this property results from their improved Δ*δ*_ab_ values, giving a steepest slope around p*K*_a_s close to that of P_i_.

## 3. Materials and Methods

### 3.1. Chemistry

All reagents and solvents were of the highest grade available from Sigma-Aldrich, Fluka, SDS, or Acros, and were used without further purification, except for 2-methyl-1-pyrroline (from Acros Organics, Halluin, France), which was distilled under reduced pressure (20 mm Hg, bp: 50 °C) before use. Diethyl H-phosphonate (**2a**), dimethyl H-phosphonate (**2g**), di(isopropyl) H-phosphonate (**2i**), and di-*n*-butyl H-phosphonate (**2j**) were from Acros. The dialkyl H-phosphonates **2b**–**f** and the aminophosphonates **1b**–**f** belonging to the CyDEPMPHs family were synthesized and purified as previously described [39]. DEPMPH (**1a**) [19], and its analogue **1i** [49], and dialkyl H-phosphonates **2h** and **2n**, and aminophosphonates **1g**, **1h**, and **1n** [29] were synthesized and purified as previously described. Reactions were followed by thin layer chromatography (TLC) on Merck-Kieselgel 60 F254 precoated silica gel plates, and the spots were visualized by staining with phosphomolybdic acid. NMR spectra (^1^H NMR at 300 MHz, ^13^C NMR at 75.5 MHz and ^31^P NMR at 121.5 MHz) were recorded on a Bruker AVL 300 spectrometer. Chemical shifts (*δ*) are expressed in ppm (parts per million) relative to internal tetramethylsilane (^1^H and ^13^C) or external 85% H_3_PO_4_ (^31^P), and coupling constants *J* are given in Hz. The abbreviations s, d, t, m and q refer to singlet, doublet, triplet, multiplet and quartet signals, respectively. Melting points were determined using a B-540 Buchi apparatus and are uncorrected. Elemental analyses were performed using a Thermo Finnigan EA-1112 analyzer and were within 0.2% of theoretical values. Column chromatography was performed on Merck silica gel 60 (230~400 mesh).

#### 3.1.1. Synthesis of Dialkyl H-Phosphonates **2k**–**m**, **2o**, and **2p**

Water (1.1 g, 61 mmol) was added to HMPT (10 g, 61 mmol) in refluxing anhydrous THF (20 mL) under argon atmosphere and the mixture was stirred at reflux for 2 h. Then the corresponding alcohol (122 mmol) was added and the mixture was refluxed for another 2 h. Afterwards, the mixture was concentrated under reduced pressure to give the corresponding dialkyl H-phosphonate **2k**–**m, 2o**, and **2p** in high yields (95–98%) and a purity of >95% determined by ^31^P NMR. ^31^P NMR (121.5 MHz, CDCl_3_) *δ* **2k**, 8.42; **2l**, 8.05; **2m**, 7.80; **2o**, 9.39; **2p**, 9.53.

#### 3.1.2. General Procedure for Synthesis of Compounds **1j**–**m**, **1o**, and **1p**

A mixture of 2-methyl-1-pyrroline (1.1 eq) and the corresponding dialkyl H-phosphonate (1 eq) was stirred at room temperature until completion. TLC or ^31^P NMR were used to follow the reaction. The mixture was poured into water (aminophosphonate final concentration, ~1.8 M) and slowly acidified to pH 3 with concentrated HCl. The aqueous layer was extracted with TBME (3 × volume of water), basified with NaOH pellets to pH 9–10, and extracted with CH_2_Cl_2_ (4 × volumes of water). The combined organic phases were dried over MgSO_4_, filtered and concentrated under reduced pressure. The residue was purified by SiO_2_ column chromatography with a CH_2_Cl_2_:EtOH mixture as eluent to yield the expected aminophosphonates.

*Di-n-butyl (2-methylpyrrolidin-2-yl)phosphonate* (**1j**). Title compound was obtained by stirring 2-methyl-1-pyrroline (10 g, 120 mmol) with commercially available di-*n*-butyl H-phosphonate (21.1 g, 109 mmol) for 10 days. Flash chromatography using a CH_2_Cl_2_:EtOH, 90:10, *v:v* as eluent yielded **1j** as a yellow oil (22.3 g, 67%); ^1^H NMR (300 MHz, CDCl_3_) *δ* 4.06–3.97 (m, 4H, 2 × O-CH_2_), 3.04–2.88 (m, 2H, 5-CH_2_), 2.22–2.10 (m, 1H, 3-C(H)H), 1.83–1.70 (m, 4H, NH, 3-C(H)H, 4-CH_2_), 1.64–1.51 (m, 5H, 2 × O-CH_2_-CH_2_ and 3-C(H)H), 1.37–1.28 (q, *J* = 6.0 Hz, 4H, 2 × CH_2_-CH_3_), 1.27 (d, *J* = 15.0 Hz, 3H, 2-C(CH_3_)), 0.87 (t, *J* = 6.0 Hz, 6H, O-(CH_2_)_3_-CH_3_); ^13^C NMR (75.5 MHz, CDCl_3_) *δ* 66.0 (d, *J* = 8.2 Hz, O-CH_2_), 65.8 (d, *J* = 8.2 Hz, O-CH_2_), 59.2 (d, *J* = 163.8 Hz, 2-C), 47.0 (d, *J* = 7.5 Hz, 5-C), 34.3 (d, *J* = 3.0 Hz, 3-C), 32.7 (d, *J* = 2.5 Hz, O-CH_2_-CH_2_), 32.6 (d, *J* = 2.5 Hz, O-CH_2_-CH_2_), 25.6 (d, *J* = 4.5 Hz, 4-C), 24.2 (d, *J* = 6.0 Hz, 2-C(CH_3_)), 18.7 (2C, CH_2_-CH_3_), 13.5 (2 × O-(CH_2_)_2_-CH_3_); ^31^P NMR (121.5 MHz, CDCl_3_) *δ* 31.03. Elemental analysis calcd (%) for C_13_H_28_NO_3_P: C 56.30, H 10.18, N 5.05; found: C 54.72, H 10.10, N 5.01.

*Diisobutyl (2-methylpyrrolidin-2-yl)phosphonate* (**1k**). Title compound was obtained by stirring 2-methyl-1-pyrroline (5.5 g, 66 mmol) with commercially available diisobutyl H-phosphonate **2k** (11.8 g, 60 mmol) for 7 days. Flash chromatography using a CH_2_Cl_2_:EtOH, 90:10, *v:v* as eluent yielded **1k** as a yellow oil (11.8 g, 70%); ^1^H NMR (300 MHz, CDCl_3_) *δ* 3.54–3.43 (m, 4H, 2 × O-CH_2_), 2.73–2.57 (m, 2H, 5-CH_2_), 1.95–1.88 (m, 1H, 3-C(H)H), 1.63–1.24 (m, 6H, NH, 3-C(H)H, 4-CH_2_, 2 × CH(CH_3_)_2_), 0.98 (d, *J* = 15.0 Hz, 3H, 2-C(CH_3_)), 0.60–0.57 (d, *J* = 6.0 Hz, 12H, 2 × CH(CH_3_)_2_); ^13^C NMR (75.5 MHz, CDCl_3_) *δ* 71.4 (d, *J* = 7.5 Hz, O-CH_2_), 71.1 (d, *J* = 7.5 Hz, O-CH_2_), 58.8 (d, *J* = 165.3 Hz, 2-C), 46.2 (d, *J* = 7.5 Hz, 5-C), 32.9 (d, *J* = 2.5 Hz, 3-C), 28.4 (2 × CH(CH_3_)_2_), 24.5 (d, *J* = 4.5 Hz, 4-C), 23.5 (d, *J* = 6.0 Hz, 2-C(CH_3_)), 17.8 (2 × CH(CH_3_)_2_); ^31^P NMR (121.5 MHz, CDCl_3_) *δ* 31.17. Elemental analysis calcd (%) for C_13_H_28_NO_3_P: C 56.30, H 10.18, N 5.05; found: C 55.06, H 10.56, N 4.96.

*Dipentyl (2-methylpyrrolidin-2-yl)phosphonate* (**1l**). Title compound was obtained by stirring 2-methyl-1-pyrroline (2.5 g, 30 mmol) with dipentyl H-phosphonate **2l** (6 g, 27.3 mmol) for 7 days. Flash chromatography using a CH_2_Cl_2_:EtOH, 90:10, *v:v* as eluent yielded **1l** as a yellow oil (4.2 g, 49%); ^1^H NMR (300 MHz, CDCl_3_) *δ* 4.07–3.99 (m, 4H, 2 × O-CH_2_), 3.07–2.91 (m, 2H, 5-CH_2_), 2.22–2.18 (m, 1H, 3-C(H)H), 1.90–1.52 (m, 8H, 2 × OCH_2_CH_2_, NH, 3-C(H)H, 4-CH_2_), 1.30 (m, 8H, 2 × CH_2_CH_2_CH_3_), 1.30 (d, *J* = 15.0 Hz, 3H, 2-C(CH_3_)), 0.86 (t, *J* = 9.0 Hz, 6H, 2 × CH_2_CH_3_); ^13^C NMR (75.5 MHz, CDCl_3_) *δ* 66.4 (d, *J* = 7.5 Hz, O-CH_2_), 66.1 (d, *J* = 7.5 Hz, O-CH_2_), 59.6 (d, *J* = 164.6 Hz, 2-C), 47.1 (d, *J* = 7.5 Hz, 5-C), 34.6 (d, *J* = 2.5 Hz, 3-C), 30.4 (d, *J* = 4.5 Hz, 4-C), 27.6 (2C, CH_2_CH_2_CH_3_), 25.7 (d, *J* = 4.5 Hz, 2C, OCH_2_CH_2_), 24.3 (d, *J* = 6.8 Hz, 2-C(CH_3_)), 22.2 (2 × CH_2_CH_3_), 13.9 (2C, CH_2_CH_3_); ^31^P NMR (121.5 MHz, CDCl_3_) *δ* 30.95. Elemental analysis calcd (%) for C_15_H_32_NO_3_P: C 58.99, H 10.56, N 4.59; found: C 56.10, H 10.35, N 3.90.

*Diisopentyl (2-methylpyrrolidin-2-yl)phosphonate* (**1m**). Title compound was obtained by stirring 2-methyl-1-pyrroline (4.4 g, 53 mmol) with diisopentyl H-phosphonate **2m** (10.8 g, 48.2 mmol) for 7 days. Flash chromatography using a CH_2_Cl_2_:EtOH, 90:10, *v:v* as eluent yielded **1m** as a yellow oil (4.2 g, 49%); ^1^H NMR (300 MHz, CDCl_3_) *δ* 4.07–3.99 (m, 4H, 2 ×O-CH_2_), 3.07–2.91 (m, 2H, 5-CH_2_), 2.22–2.18 (m, 1H, 3-C(*H*)H), 1.90–1.52 (m, 8H, 2 × OCH_2_C*H*_2_, NH, 3-C(*H*)H, 4-CH_2_), 1.30 (m, 8H, 2 × C*H*_2_C*H*_2_CH_3_), 1.30 (d, *J* = 15.0 Hz, 3H, 2-C(CH_3_)), 0.86 (t, *J* = 9.0 Hz, 6H, 2 × CH_2_C*H*_3_); ^13^C NMR (75.5 MHz, CDCl_3_) *δ* 66.4 (d, *J* = 7.5 Hz, O-*C*H_2_), 66.1 (d, *J* = 7.5 Hz, O-*C*H_2_), 59.6 (d, *J* = 164.6 Hz, 2-C), 47.1 (d, *J* = 7.5 Hz, 5-C), 34.6 (d, *J* = 2.5 Hz, 3-C), 30.4 (d, *J* = 4.5 Hz, 4-C), 27.6 (2C, *C*H_2_CH_2_CH_3_), 25.7 (d, *J* = 4.5 Hz, 2C, OCH_2_*C*H_2_), 24.3 (d, *J* = 6.8 Hz, 2-C(*C*H_3_)), 22.2 (2 × *C*H_2_CH_3_), 13.9 (2C, CH_2_*C*H_3_); ^31^P NMR (121.5 MHz, CDCl_3_) *δ* 30.95. Elemental analysis calcd (%) for C_15_H_32_NO_3_P: C 58.99, H 10.56, N 4.59; found: C 56.10, H 10.35, N 3.90.

*Di(2-ethoxyethyl)(2-methylpyrrolidin-2-yl)phosphonate* (**1o**). Title compound was obtained by stirring 2-methyl-1-pyrroline (2.5 g, 30 mmol) with diethoxyethyl H-phosphonate **2o** (6.2 g, 27.3 mmol) for 1 day. Flash chromatography using a CH_2_Cl_2_:EtOH, 96:4, *v:v* as eluent yielded **1o** as a yellow oil (6.1 g, 73%); ^1^H NMR (300 MHz, CDCl_3_) *δ* 4.23–4.11 (m, 4H, 2 × P-O-C*H*_2_), 3.66–3.62 (t, *J* = 6.0 Hz, 4H, 2 × C*H*_2_OCH_2_CH_3_), 3.57–3.50 (q, *J* = 6.0 Hz, 4H, 2 × C*H*_2_CH_3_), 3.02–2.90 (m, 2H, 5-CH_2_), 2.25–2.17 (m, 1H, 3-C(*H*)H), 1.90–1.53 (m, 4H, 3-C(*H*)H, 4-CH_2_, NH), 1.31 (d, *J* = 15.0 Hz, 2-C(CH_3_)), 1.13 (t, *J* = 9.0 Hz, 6H, 2 × CH_2_C*H*_3_); ^13^C NMR (75.5 MHz, CDCl_3_) *δ* 69.7 (d, *J* = 5.3 Hz, 2 × *C*H_2_OCH_2_CH_3_), 66.5 (2 × *C*H_2_CH_3_), 65.2 (d, *J* = 7.5 Hz, P-O-CH_2_), 65.3 (d, *J* = 7.5 Hz, P-O-CH_2_), 59.7 (d, *J* = 163.8 Hz, 2-C), 46.9 (d, *J* = 6.8 Hz, 5-C), 34.6 (d, *J* = 3.8 Hz, 3-C), 25.6 (d, *J* = 3.8 Hz, 4-C), 24.2 (d, *J* = 6.8 Hz, 2-C(*C*H_3_)), 15.1 (2C, CH_2_*C*H_3_); ^31^P NMR (121.5 MHz, CDCl_3_) *δ* 31.65. Elemental analysis calcd (%) for C_13_H_28_NO_3_P: C 50.48, H 9.12, N 4.53; found: C 49.16, H 9.03, N 4.39.

*Di(2,5,8,11-tetraoxatridecan-13-yl)(2-methylpyrrolidin-2-yl)phosphonate* (**1p**). Title compound was obtained by stirring 2-methyl-1-pyrroline (0.6 g, 7.5 mmol) with di(2,5,8,11-tetraoxatridecan-13-yl) H-phosphonate **2p** (3.2 g, 6.8 mmol) for 1 day. Flash chromatography using a CH_2_Cl_2_:EtOH, 96:4, *v:v* as eluent yielded **1q** as a yellow oil (2.7 g, 71%); ^1^H NMR (300 MHz, CDCl_3_) *δ* 4.30–4.12 (m, 4H, 2 × POCH_2_), 3.71–3.65 (m, 4H, 2 × OCH_2_), 3.65–3.58 (m, 20H, 10 × OCH_2_), 3.55–3.49 (m, 4H, 2 × OC*H*_2_), 3.33 (6H, 2 × OCH_3_), 3.10–2.91 (m, 2H, 5-CH_2_), 2.28–2.17 (m, 1H, 3-C(*H*)H), 1.90–1.52 (m, 4H, 3-C(*H*)H, 4-H, NH), 1.32 (d, *J* = 15.0 Hz, 2-C(CH_3_)); ^13^C NMR (75.5 MHz, CDCl_3_) *δ* 72.7 (OCH_2_), 71.8 (OCH_2_), 70.5 (OCH_2_), 70.4 (OCH_2_), 70.3 (OCH_2_), 70.1 (OCH_2_), 65.3 (d, *J* = 7.5 Hz, POCH_2_), 65.2 (d, *J* = 7.5 Hz, POCH_2_), 59.7 (d, *J* = 164.6 Hz, 2-C), 58.9 (2C, OCH_3_), 46.9 (d, *J* = 7.5 Hz, 5-C), 34.5 (d, *J* = 2.3 Hz 3-C), 25.5 (d, *J* = 4.5 Hz, 4-C), 23.0 (d, *J* = 6.8 Hz, 2-C(*C*H_3_)); ^31^P NMR (121.5 MHz, CDCl_3_) *δ* 29.52. Elemental analysis calcd (%) for C_23_H_48_NO_11_P: C 50.63, H 8.87, N 2.57; found: C 47.67, H 8.84, N 2.37.

### 3.2. X-ray Crystallography

CyDEPMPHs were dissolved in the minimum of CH_2_Cl_2_, recrystallized from TBME, and dried in a vacuum to give single crystals suitable for X-ray diffraction studies. Intensities were collected at 293 K on a Nonius Kappa CCD diffractometer (Bruker) using graphite-monochromated Mo_Kα_ radiation (λ = 0.71073 Å).

Crystal data for **1b**: C_8_H_16_NO_3_P *M* = 205.20, monoclinic space group *P*-21 c, Hall group *-P 2ybc*, *a* = 12.3308(2), *b* = 13.7792(2), *c* = 15.2243(2) Å, *β* = 126.3761(7)°, *V* = 2082.69(5) Å^3^, *Z* = 4.

Crystal data for *trans*-**1c**: C_9_H_18_NO_3_P *M* = 219.21, monoclinic space group *P*-21 c, Hall group *-P 2ybc*, *a* = 6.6716(1), *b* = 12.6291(2), *c* = 13.6892(3) Å, *β* = 92.9872(8)°, *V* = 1151.83(4) Å^3^, *Z* = 4.

Crystal data for **1d**: C_10_H_20_NO_3_P *M* = 233.24, monoclinic space group *P*-21 c, Hall group *-P 2ybc*, *a* = 10.5502(5), *b* = 11.1665(4), *c* = 11.4207(2) Å, *V* = 1214.42(9) Å^3^, *Z* = 4.

Crystal data for **1e**: C_12_H_24_NO_3_P *M* = 261.29, orthorhombic space group *P*bca, Hall group *-P 2ac 2ab*, *a* = 13.3114(2), *b* = 10.4734(2), *c* = 20.0050(2) Å, *V* = 2789.01(9) Å^3^, *Z* = 8.

Crystal data for **1f**: C_12_H_24_NO_3_P *M* = 261.29, triclinic space group *P*-1, Hall group -*P 1, a* = 6.5802(5), *b* = 7.9200(9), *c* = 14.228(1) Å, *V* = 699.37(11) Å^3^, *Z* = 2.

CCDC 794184, 794158, 794208, 794102, 793,993 contains the supplementary crystallographic data of **1b**, *trans*-**1c**, **1d**–**f**, respectively. These data are provided free of charge by The Cambridge Crystallographic Data Centre, 12 Union Road, Cambridge, UK (fax: +44 1223 336033; e-mail: deposit@ccdc.cam.ac.uk).

### 3.3. P NMR pH-Titration of Aminophosphonates ***1a-p***

All titrations were carried out at 22 °C in modified KH medium (pH 7.35), consisting of (in mM): KH_2_PO_4_, 1.2; MgSO_4_, 1.2; NaCl, 118.5; KCl, 4.8; NaHCO_3_, 25, and EDTA, 0.55 dissolved in doubly distilled deionized water. Spectra were acquired (32 scans) at 22 °C on a Bruker AMX 400 spectrometer with a 9.4 Tesla wide-bore magnet at a phosphorus frequency of 161.98 MHz. Samples were placed in 10-mm tubes. ^2^H_2_O in a small capillary was used as a lock signal for the spectrometer. Chemical shifts are given in ppm with respect to 85% external H_3_PO_4_ at 0 ppm and were plotted against pH to fit the Henderson–Hasselbach Equation (2) for NMR using nonlinear regression (Prism, GraphPad Software Inc., San-Diego, CA, USA):

(2) $$pH=pK_{a}+\log\left[\frac{\delta -\delta_{a}}{\delta_{b}-\delta }\right]$$

where *δ* is the experimental ^31^P chemical shift and the *δ_a_* and *δ_b_* correspond to the limiting chemical shift values of protonated and unprotonated amines forms, respectively. Calculated titration data (*pK_a_*, *δ_a_*, *δ_b_* and their difference (Δ*δ_ab_*)) are expressed as mean ± SD.

### 3.4. Computational Methods for [C–P–O] Angle Calculations

[C–P–O] angle calculations were performed for both acidic and basic forms of the aminophosphonates. To improve the accuracy of the calculations for compounds **1a**–**p**, compounds LAP-**1**–**34**, CAP-**1**–**10**, APA-**1**–**5** (Table S1), and 6 amines (i.e., dimethylamine, diethylamine, diisopropylamine, piperidine, pyrrolidine, tetramethylpiperidine) were included to the pooled molecules. The angle calculations were performed using Gaussian16 software [50] after geometry optimization at B3LYP/6-31G(d) level of theory. To obtain the most stable conformation of each molecule before DFT optimization, a simulated annealing calculation at the AM1 level with the Ampac 11 [51] software package was performed. The optimizations were achieved with a maximum time of 12 h. A check for the absence of imaginary frequencies and a gradient close to zero was performed. All calculations were performed in water solvent with the Polarizable Continuum Model (PCM) [52]. Data (in °) are expressed as the differences in [C–P–O] angles between the acidic and basic structures (Δ[C–P–O]_ab_).

### 3.5. Cell Culture, Cytotoxicity Assays, and pH Assessment in Amoebae

DMEM, GlutaMax™, and phosphate-buffered saline (PBS) were obtained from Gibco Life Technologies Inc. (Thermo Fisher Scientific, Illkirch, France). FBM and growth factors (FGM; Clonetics FGM-2 Bullet Kit) were from Lonza (Amboise, France). A549 (ATCC CCL-185; LGC Standards, Molsheim, France) and NHLF (Lonza) cells were routinely maintained in [DMEM + GlutaMax] and [FBM + FGM], respectively, as described previously [53,54]. After reaching 90% confluence, cells were harvested for subculture. Cells were trypsinized, seeded in 96-well microplates (density, 2.5 × 10^4^ cells/well) and incubated at 37 °C in a humidified atmosphere with 5% CO_2_ to reach ~80% confluence in the appropriate medium. The medium was renewed and cells were exposed for 48 h to the tested compounds (1–1000 µg/mL) or DMSO (0.2%) added medium (control). Afterwards, cells were washed twice with PBS 1X (+/+) for cytotoxicity analysis. Supernatant medium samples were kept to evaluate cytosolic LDH release using a commercial kit (Biolabo, Maisy, France). To estimate the total LDH content, a control measurement was performed for each set of experiments by treating cells with 1% Triton X-100 to induce a total LDH release in the supernatant and induce a 100% loss of viability.

The FMCA and MTT assays were carried out as described [55]. Intracellular ATP content was assayed using a luciferin–luciferase reagent (Biofax A^®^; Yelen Analytics, Marseille, France; http://www.yelen-analytics.com (accessed on 10 January 2021)) according to [55]. IC_50_ values, defined as the concentration of test compounds resulting in 50% cell viability after 48 h, were calculated from concentration–response curves (PrismSoftware) and are expressed as mean ± SD (standard deviation relative to the mean from 3–10 independent experiments).

### 3.6. Dictyostelium Discoideum Cells Cultures and ^31^P NMR

*D. discoideum* amoebae, axenic strain (ATCC 24397), were cultured aerobically as previously described [21,29]. Briefly, cells were harvested in their exponential growth phase, washed with ice-cold 20 mM 2-morpholinoethanesulfonic acid sodium salt (MES-Na) buffer and then suspended (3 × 10^8^ cells/mL) at 20 °C under O_2_ bubbling in MES-Na (20 mM), 6% ^2^H_2_O and 5 µL of Antifoam 289 (Sigma-Aldrich, Saint Quentin Fallavier, France) to a final volume of 20 mL (pH 6.5). Afterwards, **1a** or **1b** were added (4 mM final concentration) to the cell medium and incubation was prolonged for up to 30 min at 20 °C.

Incubation samples were then placed in 25-mm NMR tubes and an ^2^H_2_O sample, placed in a small capillary, was used as a lock signal for the NMR spectrometer. ^31^P NMR spectra were routinely recorded during cell incubation at 20 °C on a Bruker AMX 400 spectrometer with a 9.4 T wide-bore magnet at a phosphorus frequency of 161.98 MHz. Chemical shifts are given in ppm with respect to 85% external H_3_PO_4_ at 0 ppm and to 50 mM methylene diphosphonate (pH 8.9), placed in a capillary and used as an additional standard at 16.4 ppm. Spectral acquisitions were carried out using a 60° (10 µs) pulse width, a 0.28 s acquisition time, a 0.72 s repetition delay, and gated Waltz proton decoupling [21]. Data were stored during 120 min as 300-scan (5 min) or 900-scan (15 min) blocks. Gaussian line broadening (GB = 0.05, LB = –5 Hz) was applied prior to Fourier transformation. The extracellular and intracellular distribution of the probe was determined by the respective areas of the corresponding resonances.

### 3.7. Statistics

Titration and biological values are expressed as mean ± SD. Differences were analyzed using a one-way analysis of variance (ANOVA) followed by *a posteriori* Newman–Keuls test. Intergroup differences were considered to be significant at *p* < 0.05.

## 4. Conclusions

A novel series of fifteen α-aminophosphonates have been synthesized and screened for their ^31^P NMR properties in the probing of subtle pH changes (as low as 0.05 pH units) occurring in normal or acidotic cells. The new compounds, obtained in only two or three synthetic steps, showed the following enhanced properties: (i) chemical shifts in the 18–36 ppm range distinct from those of phosphorus metabolites; (ii) near-neutral p*K*_a_s (6.3–7.0); (iii) a high NMR sensitivity (Δ*δ*_ab_ ranging 9.2–10.6 ppm) that can be modulated by adjusting the substituents and steric effects around the phosphorus atom; (iv) no cytotoxic effect for most of them in the concentration range used for biological NMR applications (up to 20 mM); (v) the ability to penetrate the compartments of interest (i.e., intra- and extracellular media); (vi) protonated and non-protonated forms in fast exchange on the NMR timescale, thus providing a single signal for each compartment. The hit compound **1b** was applied successfully to accurately measure a trans-sarcolemmal pH gradient in *D. discoideum*. It is believed this aminophosphonate may be widely used to study the proton exchange dynamics between cellular compartments.

## Figures and Tables

**Table 1:**
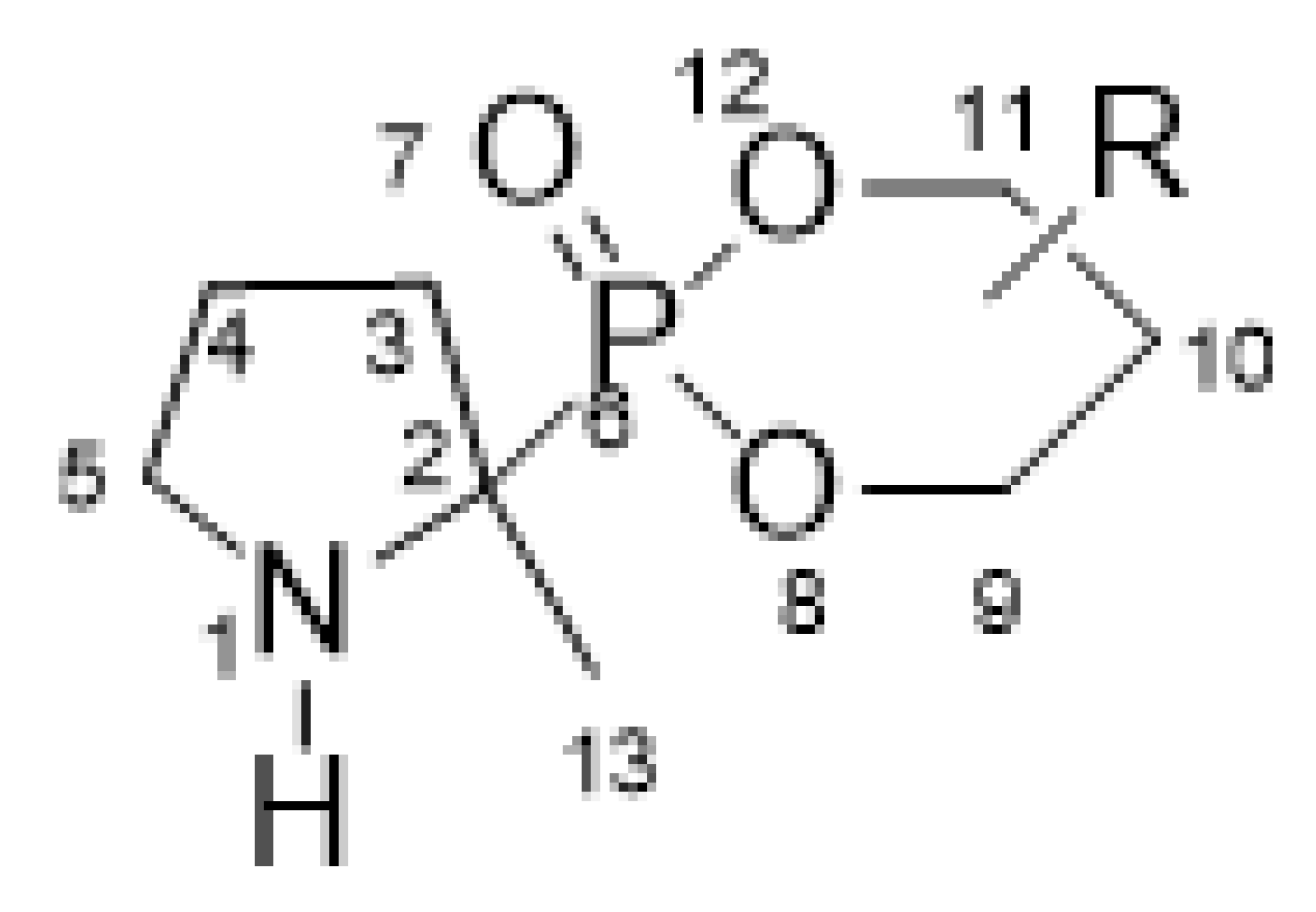
General Structure, Atoms Numbering and Selected X-Ray Crystallographic Data of CyDEPMPHs ^a^.

	1b	*trans*-1c	1d	1e	1f
*Bond length (Å)*					
N(1)–H	0.941(0)	1.058(0)	1.053(0)	1.004(0)	0.990(0)
N(1)–C(2)	1.481(2)	1.482(3)	1.485(3)	1.482(3)	1.486(3)
N(1)–C(5)	1.463(4)	1.452(3)	1.478(3)	1.480(3)	1.461(3)
C(2)–P(6)	1.810(2)	1.813(2)	1.814(2)	1.826(2)	1.815(2)
P(6)–O(7)	1.466(2)	1.465(2)	1.462(2)	1.466(2)	1.465(2)
P(6)–O(8)	1.570(2)	1.579(2)	1.573(1)	1.578(2)	1.575(1)
P(6)–O(12)	1.576(2)	1.571(1)	1.579(1)	1.581(2)	1.561(2)
*Distance length (Å)*					
H–O(7)	4.559(1)	4.560(2)	4.589(0)	4.644(1)	4.658(1)
*Bond angle (°)*					
C(5)–N(1)–C(2)	108.1(2)	108.3(2)	106.4(2)	109.0(2)	106.7(2)
N(1)–C(2)–C(3)	105.7(2)	105.2(2)	106.7(1)	105.0(2)	106.1(2)
N(1)–C(2)–P(6)	107.9(1)	108.9(1)	108.6(1)	108.6(1)	110.0(2)
N(1)–C(2)–C(13)	111.7(2)	111.6(2)	110.5(1)	111.7(2)	110.6(2)
C(2)–P(6)–O(7)	112.9(1)	112.0(1)	112.3(9)	113.1(1)	111.5(1)
C(2)–P(6)–O(12)	107.5(9)	107.1(9)	107.4(8)	107.7(9)	109.1(1)
C(2)–P(6)–O(8)	106.9(9)	108.2(1)	1080(8)	107.7(9)	105.1(9)
C(13)–C(2)–P(6)	109.4(1)	110.0(2)	108.8(1)	108.4(1)	108.7(2)
*Dihedral angle (°)*					
C(5)–N(1)–C(2)–C(3)	−0.5(2)	−1.0(2)	−9.0(2)	0.4(2)	10.9(2)
C(5)–N(1)–C(2)–P(6)	117.7(2)	−117.9(2)	109.3(2)	−117.8(2)	106.7(2)
N(1)–C(2)–C(3)–C(4)	5.6(0)	24.1(2)	−12.3(2)	−20.2(2)	12.9(2)
H– N(1)–C(2)–P(6)	−122.6(2)	127.9(0)	−132.6(1)	138.8(1)	119.7(1)
N(1)–C(2)–P(6)–O(7)	−174.1(1)	175.8(1)	−175.8(1)	177.2(1)	170.8(1)
N(1)–C(2)–P(6)–O(12)	−92.2(0)	53.2(2)	−51.6(1)	53.9(1)	46.9(2)
N(1)–C(2)–P(6)–O(8)	7.87(1)	−60.4(2)	61.8(1)	−58.9(1)	–66.5(2)
C(13)–C(2)–P(6)–O(7)	64.2(2)	−61.5(2)	63.9(1)	−61.3(2)	–68.0(2)

^a^ Values in parentheses are estimated standard deviations.

**Table 2:** ^31^P NMR Parameters and p*K*_a_ Values ^a^ in KH Medium at 22 °C.

Compounds	p*K*_a_	*δ_a_* (ppm)	*δ_b_* (ppm)	Δ*δ*_ab_ (ppm)	*T*_1_ (s)
**P**_i_	6.72 ± 0.11	0.81 ± 0.02	3.32 ± 0.03	2.51 ± 0.03	10.50
**1a**	7.01 ± 002	23.08 ± 0.06	32.85 ± 0.06	9.77 ± 0.11	5.40
*CyDEPMPHs family*					
**1b**	6.45 ± 0.01	20.65 ± 0.05	31.29 ± 0.04	10.64 ± 0.09	6.00
***trans*-1c**	6.45 ± 0.02	19.07 ± 0.11	29.95 ± 0.08	10.58 ± 0.19	5.11
***cis*-1c**	6.54 ± 0.03	20.60 ± 0.16	31.08 ± 0.12	10.48 ± 0.28	4.77
**1d**	6.22 ± 0.01	19.63 ± 0.08	30.33 ± 0.06	10.70 ± 0.14	4.51
**1e**	6.28 ± 0.01	20.58 ± 0.05	31.32 ± 0.05	10.74 ± 0.10	4.45
**1f**	6.79 ± 0.01	18.34 ± 0.03	27.69 ± 0.03	9.35 ± 0.06	4.06
*Crowded family*					
**1g**	6.70 ± 0.01	26.29 ± 0.05	35.89 ± 0.04	9.60 ± 0.09	7.96
**1h**	6.89 ± 0.01	23.85 ± 0.04	33.39 ± 0.04	9.55 ± 0.08	5.06
**1i**	6.97 ± 0.02	21.44 ± 0.10	31.30 ± 0.10	9.86 ± 0.20	4.70
**1j**	6.88 ± 0.01	23.85 ± 0.06	33.45 ± 0.05	9.60 ± 0.11	4.27
**1k**	6.78 ± 0.04	23.69 ± 0.20	33.03 ± 0.18	9.34 ± 0.38	4.16
**1l**	6.71 ± 0.01	23.78 ± 0.07	33.35 ± 0.07	9.57 ± 0.14	3.90
**1m**	6.76 ± 0.02	23.93 ± 0.10	33.37 ± 0.10	9.44 ± 0.20	3.62
**1n**	6.71 ± 0.01	23.57 ± 0.06	32.76 ± 0.06	9.19 ± 0.12	3.91
**1o**	6.62 ± 0.01	24.09 ± 0.07	33.88 ± 0.06	9.79 ± 0.13	3.75
**1p**	6.63 ± 0.01	24.10 ± 0.09	33.84 ± 0.07	9.74 ± 0.16	2.64

^a^ Values are means ± SD (*n* = 3–10); *δ*_a_ and *δ*_b_, limiting chemical shifts of the protonated and unprotonated form, respectively; Δ*δ*_ab_ = *δ*_a_ − *δ*_b_.

**Table 3:** Cytotoxicity of α-Aminophosphonates Against A549 Cells and Normal Human Lung Fibroblasts (NHLF) ^a^, and their Calculated Lipophilicities.

Compounds	IC_50_ (mM) ^b^						AlogP ^c^
	FMCA		MTT		ATP		
	A549	NHLF	A549	NHLF	A549	NHLF	
**1a**	122 ± 8	112 ± 7	118 ± 11	110 ± 8	119 ± 7	109 ± 10	1.04
*CyDEPMPHs family*							
**1b**	95± 8	89 ± 5	92 ± 8	79 ± 6	85 ± 9	70 ± 7	0.26
*trans* **-1c**	80 ± 5	72 ± 7	76 ± 5	70 ± 3	69 ± 7	62 ± 6	0.54
*cis* **-1c**	77 ± 5	70 ± 5	78 ± 7	71 ± 9	67 ± 5	61 ± 5	0.54
**1d**	74 ± 9	69 ± 6	62 ± 5 *	58 ± 8 *	63 ± 5 *	56 ± 8	0.77
**1e**	70 ± 7 *	67 ± 8 *	65 ± 6 *	67 ± 7 *	62 ± 5 *	55 ± 5 *	1.51
**1f**	44 ± 6 *^,†^	35 ± 7 *^,†^	52 ± 9 *^,†^	45 ± 4 *^,†^	53 ± 4 *^,†^	47 ± 6 *^,†^	1.73
*Crowded family*							
**1g**	118 ± 9	104 ± 6	111 ± 3	97 ± 4	107 ± 9	92 ± 9	0.25
**1h**	101 ± 4	95 ± 7	97 ± 4	89 ± 2	98 ± 5	87 ± 5	1.65
**1i**	103 ± 3	98 ± 3	95 ± 4	90 ± 3	96 ± 4	89 ± 6	1.46
**1j**	18 ± 7 *^,§^	16 ± 7 *^,§^	30 ± 8 *^,§^	21 ± 7 *^,§^	31 ± 7 *^,§^	20 ± 8 *^,§^	2.31^ §^
**1k**	17 ± 6 *^,§^	14 ± 6 *^,§^	39 ± 5 *^,§^	29 ± 8 *^,§^	29 ± 7 *^,§^	14 ± 8 *^,§^	2.20^ §^
**1l**	11 ± 8 *^,§^	7 ± 6 *^,§^	21 ± 7 *^,§^	14 ± 5 *^,§^	23 ± 7 *^,§^	12 ± 8 *^,§^	3.31^ §^
**1m**	10 ± 4 *^,§^	8 ± 7 *^,§^	15 ± 4 *^,§^	11 ± 2 *^,§^	14 ± 3 *^,§^	13 ± 4 *^,§^	3.03^ §^
**1n**	12 ± 3 *^,§^	9 ± 4 *^,§^	16 ± 2 *^,§^	10 ± 2 *^,§^	17 ± 7 *^,§^	13 ± 6 *^,§^	2.92^ §^
**1o**	11 ± 5 *^,§^	7 ± 4 *^,§^	14 ± 3 *^,§^	11 ± 2 *^,§^	16 ± 3 *^,§^	12 ± 4 *^,§^	0.90^ §^
**1p**	13 ± 3 *^,§^	10 ± 4 *^,§^	16 ± 2 *^,§^	11 ± 2 *^,§^	20 ± 4 *^,§^	17 ± 4 *^,§^	0.25^ §^

^a^ Cells seeded at 2.5 × 10^4^ cells/well in DMEM (A549) or FBM (NHLF) until confluence were then incubated for 48 h at 37 °C with test compounds (0.01–100 mM) in culture medium + 0.2% DMSO (taken as vehicle). Data are means ± SD of 3–10 independent experiments made in triplicate for at least four concentrations. ^b^ IC_50_ defined as the concentration of compound resulting in 50% loss of cell viability after 48 h and calculated from concentration–response curves. ^c^ Calculated using the ALOGPS 2.1 software (available at Virtual Computational Chemistry Laboratory: www.vcclab.org/lab/alogps/ accessed on 25 July 2021). Statistics: one-way-ANOVA followed by the Newman–Keuls test: * *p* < 0.05 vs. **1b**, *trans***-1c,** and *cis***-1c**; ^†^ *p* < 0.05 vs. **1d** and **1e**; ^§^ *p* < 0.05 vs. **1g**, **1h**, and **1i.**

## Data Availability

The data presented in this study are available on request from the authors.

## Supplementary Materials

The following are available online at https://www.mdpi.com/article/10.3390/molecules27144506/s1, Table S1: p*K*_a_ and Δ*δ*_ab_ values in KH medium reported in the literature for various aminophosphosphorylated compounds; Table S2: Substituents types and Increments *a*_i_ for p*K*_a_ calculation using Equation (1).

## Sample Availability

Samples of the compounds are not available from the authors.
