# Supplementary material for: Novel Sterically Crowded and Conformationally Constrained α-Aminophosphonates with a Near-Neutral pKa as Highly Accurate 31P NMR pH Probes. Application to Subtle pH Gradients Determination in Dictyostelium discoideum Cells
Source: Molecules. 2022 Jul 14;27(14):4506. doi: 10.3390/molecules27144506 (PMC9320275; doi:10.3390/molecules27144506)
Supplement: Supplementary file 1 [file molecules-27-04506-s001.zip › molecules-1758835-supplementary.pdf]

Supplementary Materials

Novel Sterically Crowded and Conformationally Constrained α-Aminophosphonates with a Near-Neutral pK_a as Highly Accurate ^{31}P NMR pH Probes. Application to Subtle pH Gradients Determination in *Dictyostelium discoideum* Cells

Caroline Delehedde [1], Marcel Culcasi [1], Emilie Ricquebourg [1], Mathieu Cassien [2], Didier Siri [3], Bruno Blaive [1], Sylvia Pietri [1] and Sophie Thétiot-Laurent [1,*]

[1] Aix Marseille Univ, CNRS, ICR, UMR 7273, SMBSO, 13397 Marseille, France
[2] Yelen Analytics, 10 Boulevard Tempête, 13820 Ensuès-la-Redonne, France
[3] Aix Marseille Univ, CNRS, ICR, UMR 7273, CT, 13397 Marseille, France

Corresponding Author

S. Thétiot-Laurent, Ph.D.

Aix Marseille Univ, CNRS, ICR UMR 7273, Institut de Chimie Radicalaire

Sondes Moléculaires en Biologie et Stress Oxydant, Service 522

Facultédes Sciences de Saint-Jérôme, Avenue Escadrille Normandie-Niemen

13397 Marseille Cedex 20 - France

Tel: +33-(0)4-13-94-58-07; Fax: +33-(0)4-91-28-87-58

sophie.thetiot-laurent@univ-amu.fr

Table of contents

Table S1 .... S2

Table S2 .... S5

Supplementary References S6

Table S1. pK_a and $\Delta\delta_{ab}$ (absolute value of the difference between limiting chemical shift of the protonated and unprotonated form) values in Krebs-Henseleit (KH) medium reported in the literature for various aminophosphophosphorylated compounds.

Compound	Position of substituent	pK_a	$\Delta\delta_{ab}$ (ppm)	Ref.

Linear α-aminophosphonates (LAP)

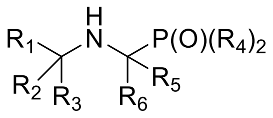

Compound	Position of substituent	pK_a	$\Delta\delta_{ab}$ (ppm)	Ref.
LAP-**1**	$R_1 = R_2 = H$; $R_3 = Et$; $R_4 = OEt$; $R_5 = R_6 = Me$	6.99	10.17	[19]
LAP-**2**	$R_1 = R_2 = H$; $R_3 = Et$; $R_4 = OEt$; $R_5 = Me$; $R_6 = 4\text{-}NO_2Ph$	5.03	8.02	[19]
LAP-**3**	$R_1 = R_2 = H$; $R_3 = Et$; $R_4 = OEt$; $R_5 = Me$; $R_6 = Ph$	5.89	8.19	[28]
LAP-**4**	$R_1 = R_2 = H$; $R_3 = Ph$; $R_4 = OEt$; $R_5 = Me$; $R_6 = Ph$	4.76	8.14	[29]
LAP-**5**	$R_1 = R_2 = H$; $R_3 = n\text{-}Pr$; $R_4 = OEt$; $R_5 = Me$; $R_6 = H$	6.92	10.17	[28]
LAP-**6**	$R_1 = R_2 = Me$; $R_3 = CH_2OH$; $R_4 = OEt$; $R_5 = Me$; $R_6 = H$	5.66	10.01	[28]
LAP-**7**	$R_1 = R_2 = R_3 = Me$; $R_4 = OEt$; $R_5 = Me$; $R_6 = H$	7.02	10.29	[28]
LAP-**8**	$R_1 = R_2 = R_3 = Me$; $R_4 = OEt$; $R_5 = i Pr$; $R_6 = H$	6.61	10.94	[19]
LAP-**9**	$R_1 = R_2 = R_3 = Me$; $R_4 = OEt$; $R_5 = t Bu$; $R_6 = H$	6.35	10.36	[43]
LAP-**10**	$R_1 = H$; R_2,R_3 = cyclohexyl; $R_4 = OEt$; R_5,R_6 = cyclohexyl	6.29	10.18	[43]
LAP-**11**	$R_1 = R_2 = R_3 = Me$; $R_4 = Me$; $R_5 = t Bu$; $R_6 = H$	6.22	6.94	[43]
LAP-**12**	$R_1 = R_2 = R_3 = Me$; $R_4 = OEt$; $R_5 = P(O)(OEt)_2$; $R_6 = Me$	3.45	7.55	[30]
LAP-**13**	$R_1 = R_2 = R_3 = Me$; $R_4 = OEt$; $R_5 = P(O)(OEt)_2$; $R_6 = H$	3.22	8.18	[19]
LAP-**14**	$R_1 = Me$; $R_2 = H$; $R_3 = Et$; $R_4 = OEt$; $R_5 = P(O)(OEt)_2$; $R_6 = Me$	3.63	7.66	[30]
LAP-**15**	$R_1 = R_2 = R_3 = Me$; $R_4 = OEt$; $R_5 = 2\text{-}Py$; $R_6 = H$	5.32	9.12	[45]
LAP-**16**	$R_1 = R_2 = H$; $R_3 = Ph$; $R_4 = OEt$; $R_5 = P(O)(OEt)_2$; $R_6 = Et$	2.48	7.67	[30]
LAP-**17**	$R_1 = R_2 = Me$; $R_3 = H$; $R_4 = OEt$; R_5,R_6 = cyclohexyl	6.35	10.18	[19]
LAP-**18**	$R_1 = Me$; $R_2 = H$; $R_3 = CO_2Me$; $R_4 = OEt$; $R_5 = R_6 = Me$	3.63	10.38	[31]
LAP-**19**	$R_1 = Ph$; $R_2 = H$; $R_3 = CO_2Me$; $R_4 = OEt$; $R_5 = R_6 = Me$	2.60	10.37	[31]
LAP-**20**	$R_1 = i Pr$; $R_2 = H$; $R_3 = CO_2Me$; $R_4 = OEt$; $R_5 = R_6 = Me$	3.35	10.55	[31]
LAP-**21**	$R_1 = R_2 = Me$; $R_3 = CO_2Et$; $R_4 = OEt$; $R_5 = t Bu$; $R_6 = H$	2.39	10.54	[31]
LAP-**22**	$R_1 = R_2 = Me$; $R_3 = CO_2Et$; $R_4 = OEt$; $R_5 = i Pr$; $R_6 = H$	2.99	10.18	[31]

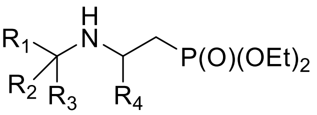

Compound	Position of substituent	pK_a	$\Delta\delta_{ab}$ (ppm)	Ref.
LAP-**23**	$R_1 = R_2 = H$; $R_3 = Et$; $R_4 = Me$	9.05	5.31	[19]

Table S1. (continued)

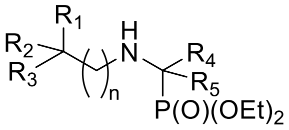

LAP-**24**	$R_1 = R_2 = H$; $R_3 = PPh_3.Br$; $R_4 = H$; $R_5 = i$Pr; n = 3	5.47	10.54	[32]
LAP-**25**	$R_1 = R_2 = H$; $R_3 = PPh_3.Br$; $R_4 = H$; $R_5 = i$Pr; n = 5	5.99	10.65	[32]
LAP-**26**	$R_1 = R_2 = H$; $R_3 = PPh_3.Br$; $R_4 = H$; $R_5 = i$Pr; n = 7	6.23	10.73	[32]
LAP-**27**	$R_1 = R_2 = H$; $R_3 = PPh_3.Br$; $R_4 = H$; $R_5 = i$Pr; n = 11	6.38	10.28	[32]
LAP-**28**	$R_1 = R_2 = H$; $R_3 = PPh_3.Br$; $R_4 = R_5 = Me$; n = 3	5.90	9.92	[32]
LAP-**29**	$R_1 = R_2 = H$; $R_3 = PPh_3.Br$; $R_4 = R_5 = Me$; n = 5	6.53	10.00	[32]
LAP-**30**	$R_1 = R_2 = H$; $R_3 = PPh_3.Br$; $R_4 = R_5 = Me$; n = 7	6.94	10.23	[32]
LAP-**31**	$R_1 = R_2 = H$; $R_3 = PPh_3.Br$; $R_4 = R_5 = Me$; n = 11	6.85	10.12	[32]
LAP-**32**	$R_1 = R_2 = H$; $R_3 = PPh_3.Br$; $R_4 = t$Bu; $R_5 = H$; n = 5	5.63	10.27	[32]

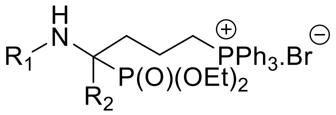

LAP-**33**	$R_1 = H$; $R_2 = Me$	5.13	9.38	[32]
LAP-**34**	$R_1 = i$Bu; $R_2 = Me$	5.39	9.84	[32]

Cyclic α-aminophosphonates (CAP)

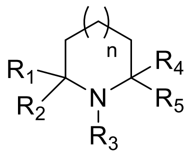

CAP-**1**	$R_1 = R_2 = H$; $R_3 = R_4 = Me$; $R_5 = P(O)(OEt)_2$; n = 0	6.47	9.80	[28]
CAP-**2**	$R_1 = R_2 = R_3 = H$; $R_4 = Me$; $R_5 = P(O)(CH_3)(OEt)$; n = 0	6.64[a]	8.32	[19]
		6.65[a]	8.49	[19]
CAP-**3**	$R_1 = R_2 = R_3 = H$; $R_4 = Ph$; $R_5 = P(O)(OEt)_2$; n = 0	5.81	8.76	[19]
CAP-**4**	$R_1 = R_2 = R_3 = H$; $R_4 = R_5 = P(O)(OEt)_2$; n = 0	3.59	7.58	[19]
CAP-**5**	$R_1 = R_2 = R_3 = H$; $R_4 = R_5 = P(O)(OiPr)_2$; n = 0	3.98	8.05	[19]
CAP-**6**	$R_1 = R_2 = R_3 = H$; $R_4 = R_5 = P(O)(OnBu)_2$; n = 0	3.44	7.51	[30]
CAP-**7**	$R_1 = R_5 = Me$; $R_2{=}R_4 = P(O)(OEt)_2$; $R_3 = H$ =; n = 0	2.42[b]	10.30	[19]
CAP-**8**	$R_1 = R_5 = Me$; $R_2{=}R_4 = P(O)(OEt)_2$; $R_3 = H$ =; n = 0	1.31[c]	8.70	[19]

Table S1. (continued)

CAP-**9**	$R_1 = R_2 = H$; $R_3 = Me$; $R_4=R_5 = P(O)(OEt)_2$; n = 0	3.63	7.60	[28]
CAP-**10**	$R_1 = R_2 = H$; $R_3 = H$; $R_4=R_5 = P(O)(OEt)_2$; n = 1	4.05	7.84	[45]

Aminophosphonic acids (APA)

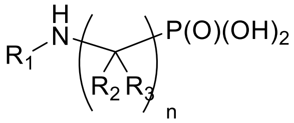

APA-**1**	$R_1 = R_2 = R_3 = H$; n = 1	5.47[d]	1.90	[22]
		10.16[e]	10.06	[22]
APA-**2**	$R_1 = R_2 = R_3 = H$; n = 2	6.31[d]	2.44	[22]
		11.05[e]	3.51	[22]
APA-**3**	$R_1 = R_2 = H$; $R_3 = Me$; n = 1	5.76[d]	1.75	[22]
		10.25[e]	9.88	[22]
APA-**4**	$R_1 = Et$; n = 0	5.76[d]	1.64	[22]
		10.30[e]	9.75	[22]
APA-**5**	$R_1 = R_2 = R_3 = H$; n = 3	6.99[d]	3.37	[22]
		11.03[e]	2.30	[22]

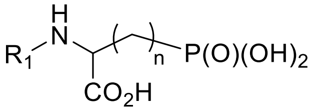

APA-**6**	$R_1 = H$; n = 1	6.15[d]	2.17	[22]
		10.55[e]	2.49	[22]
APA-**7**	$R_1 = H$; n = 2	6.85[d]	2.46	[22]
		10.69[e]	2.82	[22]

[a]undetermined diastereomers; [b]*trans*-isomer; [c]*cis*-isomer; [d]pK_{a2} of the second OH; [e]pK_{a3} of amine.
All titrations were carried out at 22 °C in modified KH medium (pH 7.35), consisting of (in mM): KH_2PO_4, 1.2; $MgSO_4$, 1.2; NaCl, 118.5; KCl, 4.8; $NaHCO_3$, 25, and EDTA, 0.55 dissolved in doubly distilled deionized water.

Table S2. Substituents Types and Increments a_i for pK_a Calculation Using: pK_a = a_0 + ($\sum a_i \times n_i$) (Equation 1 in main text)

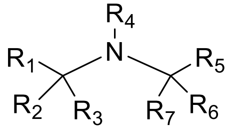

	On the N atom				On the two or three alpha carbons							
Substituent type		1st H	2nd H	Cyclic alkyl	H	Alkyl	CH_2OH	Ph	$PhNO_2$	CO_2Me	P(O)XY	*gem*P
i value	0	1	2	Reference	3	4	5	6	7	8	9	10
a_i (pH unit)	11.356	0.182	–0.688	0	–0.111	–0.091	–0.902	–0.922	–1.521	–3.332	–4.568	1.426

The contribution a_i of each substituent R_i (i = 1–7; see general structure in heading) and the constant a_0 were calculated by linear regression analysis of the experimental pK_a values at 22 °C of the following compounds: **1a–p** (see Scheme 1 in main text), LAP-**1–22**, CAP-**1–10**, APA-**1–5** (Table S1), and 6 dialkylamines (i.e., dimethylamine, pK_a = 10.64; diethylamine, pK_a = 10.98; diisopropylamine, pK_a = 11.05; piperidine, pK_a = 11.22; pyrrolidine, pK_a = 11.27; tetramethylpiperidine, pK_a = 11.72). The cyclic alkyl substituent, like in pyrrolidines, was chosen as the reference and assigned a null coefficient (i = 0). The abbreviations used are: P(O)XY, phosphonyl substituent; *gem*P incremental value to be added when a *gem* phosphorylated group is present; Ph, phenyl group. For instance, the set {n_1–n_{10}} is {1, 0, 2, 3, 0, 0, 0, 0, 1, 0} for **1a** (see Figure 1 inset in main text).

The coefficients a_0–a_6 were computed by linear regression of the pK_a values at 22 °C of 59 aminophosphonates including compounds **1a–p** and the following derivatives reported in the literature (Table S1): linear LAP-**1–22** and cyclic CAP-**1–10** aminophosphonates [19,28–31,43,45], α-, β- or γ-aminophosphophonic derivatives (APA-**1–5**) [22], and six alkylamines (i.e., dimethylamine, diethylamine, diisopropylamine, piperidine, pyrrolidine, tetramethylpiperidine [44]). It is worth noting that compounds bearing one (LAP-**23**, Table S1 [19]) or several (LAP-**24–34**, Table S1 [32]) methylene linkers were omitted from the computation since each probe would have required a coefficient per molecule that could not be associate to semi-empirical calculations. The predictive model fitted to y = 0.9735 x + 0.1383.
